# An interpretable multimodal deep learning model for physiological stress detection

**DOI:** 10.1371/journal.pone.0360314

**Published:** 2026-10-29

**Authors:** Ahmed Tibermacine, Imad Eddine Tibermacine, M’hamed Mancer, Abdelaziz Rabehi, Walid Guettala, Mustapha Habib

**Affiliations:** 1 Department of Computer Science, University of Mohamed Khider Biskra, Algeria; 2 Department of Computer, Control and Management Engineering, Sapienza University of Rome, Italy; 3 Laboratory of Telecommunications and Smart Systems, Faculty of Sciences and Technologies, University of Djelfa, Djelfa, Algeria; 4 Department of Artificial Intelligence, ELTE Eotvos Lorand University, Budapest, Hungary; 5 Division of Building Technology and Design, Department of Civil and Architectural Engineering, KTH Royal Institute of Technology, Stockholm, Sweden; Air University, PAKISTAN

## Abstract

Stress is a major contributor to physical and mental health disorders, underscoring the need for reliable and interpretable detection systems. We present an interpretable multimodal deep learning framework that integrates electrocardiogram (ECG), electrodermal activity (EDA), and respiration signals using modality-specific neural architectures complemented by feature-based classical models. To ensure robustness and generalizability, the framework was evaluated under both subject-independent and subject-dependent protocols. Results show that multimodal fusion consistently outperformed single modalities, achieving 94.8% accuracy, 93.9% F1-score, and 0.97 AUC under subject-independent validation, compared with 86–91% accuracy for individual signals. In subject-dependent experiments, performance reached 97.2% accuracy and 96.8% F1-score. Comparative analysis with recent state-of-the-art methods demonstrated the competitiveness of our approach, while ablation studies confirmed the contribution of each modality and fusion strategy. Statistical significance testing with (*p* < 0.05) further validated the reliability of these gains. Importantly, interpretability analyses using Grad-CAM and attention visualization provided physiological insights, revealing that the model emphasizes clinically meaningful regions such as ECG R-peaks and phasic EDA fluctuations. By uniting high performance, robustness, and interpretability, this work advances stress detection research and offers a physiologically grounded framework for continuous mental health monitoring.

## 1 Introduction

Stress is a pervasive condition that significantly impacts physical health, cognitive performance, and emotional well-being. Chronic exposure to stress has been associated with a wide range of adverse outcomes, including cardiovascular diseases, depression, anxiety disorders, and reduced workplace productivity [1]. With the increasing prevalence of stress in modern societies, there is a growing need for reliable, objective, and non-invasive methods for its detection and monitoring. Traditional approaches relying on self-reported questionnaires or clinical interviews, while informative, suffer from subjectivity, recall bias, and limited temporal resolution, underscoring the importance of developing physiological signal–based computational frameworks for continuous stress assessment [2,3].

Physiological signals provide a direct window into the autonomic nervous system (ANS), which plays a central role in stress responses. Among these, the electrocardiogram (ECG) reflects heart rate variability (HRV), a well-established biomarker of sympathetic–parasympathetic balance; electrodermal activity (EDA) captures changes in sweat gland activity linked to sympathetic arousal; and respiration patterns reveal alterations in breathing rhythm commonly observed under stress [4,5]. Recent advances in wearable devices and the availability of benchmark datasets such as WESAD (Wearable Stress and Affect Detection) [6] have facilitated the development of computational methods that integrate multimodal physiological signals under more naturalistic and ecologically valid conditions.

Early studies in stress detection relied mainly on hand-crafted features extracted from physiological signals, combined with classical machine learning models such as Support Vector Machines [7], Random Forests [8] and k-Nearest Neighbors [9]. Although these approaches demonstrated reasonable performance, they were heavily dependent on domain-specific feature engineering and struggled with generalization across subjects. More recently, deep learning methods have shown promise in automatically learning hierarchical representations from raw or minimally preprocessed signals [10,11]. Architectures such as Convolutional Neural Networks (CNNs), Long Short-Term Memory networks (LSTMs) and Transformer-based models have been successfully applied to stress detection, resulting in improvements in accuracy and robustness [12–14].

Despite these advances, several challenges remain. First, many existing models are evaluated in subject-dependent settings, where training and testing are performed on data from the same individuals, leading to overly optimistic performance estimates and limiting real-world applicability [9,15,16]. Second, the fusion of multimodal signals is still an open research problem. Although the combination of ECG, EDA, and respiration has shown promise, there is limited consensus on the most effective fusion strategy, whether at the raw signal, feature, or decision level [17,18]. Third, the interpretability of deep learning models is not adequately addressed [19,20]. For stress detection systems to be adopted in clinical and occupational contexts, it is essential that their predictions are physiologically meaningful and aligned with established biomarkers.

This study proposes an interpretable multimodal deep learning framework for stress detection that integrates ECG, EDA, and respiration signals through a combination of CNN, LSTM, and Transformer components, capturing both short-term temporal patterns and long-range dependencies across modalities. The model is rigorously evaluated using Leave-One-Subject-Out (LOSO) cross-validation to ensure subject-independent generalization and benchmarked against classical machine learning baselines, unimodal approaches and recent multimodal methods [21–24]. Multiple fusion strategies, ablation experiments, statistical significance tests and interpretability analyzes, including Grad-CAM and attention weight visualization, are performed to assess the contributions of complementary modalities, validate physiological plausibility, and evaluate model robustness. By addressing methodological, validation, and interpretability gaps, this work advances multimodal stress detection and supports practical applications in healthcare, workplace well-being, and mental health.

This paper is structured as follows: After reviewing related work in Section 2, Section 3 details the proposed multimodal framework. Section 4 presents the experimental results, followed by a discussion of their implications in Section 5. Finally, Section 6 concludes the study and suggests future directions.

## 2 Related work

Stress detection has extensively utilized physiological signals, with ECG being particularly informative due to its strong connection to autonomic nervous system activity. Early studies focused on hand-crafted features such as HRV [25], classified using traditional machine learning models [26–28]. Schmidt et al. [6] introduced the WESAD dataset and showed that classical models trained on engineered HRV features could achieve over 90% accuracy in binary stress versus non-stress classification.

Recent studies focus on deep learning, using CNNs and LSTMs to process raw ECG signals [29,30], capturing short-term patterns and long-term dependencies. These models often surpass traditional approaches by learning hierarchical representations directly, without manual feature engineering. In [31], zhang et al. showed that hybrid CNN–BiLSTM networks improved stress recognition from ECG alone, while Lee et al. [32] proposed an attention-based CNN-LSTM for ECG stress classification, combining spatial feature extraction, temporal modeling, and attention to highlight stress-relevant regions, improving both accuracy and interpretability [33]. Zhang et al. [34] proposed a real-time psychological stress detection method based on ECG signals using deep learning. The model automatically extracts features from ECG data to classify stress levels, achieving high accuracy and demonstrating potential for continuous monitoring in practical scenarios.

Beyond ECG-only studies, multimodal approaches have gained attention, as signals like EDA, respiration, body temperature, and accelerometer data provide complementary information for more comprehensive stress detection [35,36]. Seo et al.‘ [37] developed a deep learning model to detect work-related stress from multimodal physiological signals, improving classification accuracy through integrated sensor data. In [22], Kumar et al. proposed a hierarchical deep neural network that fused multiple physiological channels to improve classification accuracy in three-class settings (baseline, stress, amusement). Similarly, Qi et al. [20] and Vaz et al. [21] explored deep multimodal fusion strategies, reporting improved generalization across subjects compared to unimodal models [38]. More recent advances employ attention-based fusion and graph neural networks to capture inter-modality relationships. In [39], Zhu et al. applied machine learning to multimodal physiological signals from wearable devices for stress detection, achieving high accuracy in real-world settings, while Kumar et al. [40] explored multimodal fusion of EEG, ECG, and EDA for stress and emotion recognition, demonstrating the scalability of fusion strategies beyond physiological signals alone. These investigations highlight the benefit of combining ECG with EDA and respiration, where each modality captures distinct physiological responses to stress.

Cross-subject validation has also been a focal point of recent studies, as stress responses are highly individualized and subject-dependent results often fail to generalize. To address this, Li et al. [17] proposed CT-ELCAN, a cross-modal transformer architecture trained under a LOSO protocol, achieving 73.9% accuracy in three-class classification on WESAD. Albaladejo-González et al. [15], emphasized the use of transfer learning to improve the generalization of cross-dataset for stress recognition using heart rate and HRV. More recently, Vos et al. [18] investigated ensemble learning techniques by training a model on a newly synthesized dataset, demonstrating strong generalization for stress prediction using wearable devices. Mohammadi et al. [9], on the other hand, explored domain adaptation and supervised algorithms in an integrated human stress detection sensor to enhance performance across different individuals and datasets. Akbari et al. [41] further demonstrated the use of meta-learning for personalization, enabling models to quickly adapt to new subjects with limited data. In [42], Tefera et al. applied advanced denoising and meta-learning techniques to wearable sensor data, improving reliability in smart health monitoring.These studies demonstrate that robust evaluation in subject-independent settings remains a key challenge in physiological stress detection, as accuracy is typically lower compared to subject-dependent splits.

Another important area is explainability, where the goal is to validate that stress detection models rely on physiologically meaningful patterns rather than spurious correlations [43]. Zhu et al. [44] and Vos et al. [18] highlighted the need for transparent models employing attention visualization and interpretable feature attribution methods. More advanced approaches, such as Gradient-weighted Class Activation Mapping (Grad-CAM), have been adapted from computer vision to time series signals, providing insight into whether models focus on relevant cardiac cycles or EDA peaks during stress episodes [45]. Recent works by Han et al. [46] proposed a multimodal framework with explainable AI for early workplace stress detection, integrating multiple signals to improve accuracy and interpretability, while Park et al. [47] combined SHAP and attention mechanisms to improve the interpretability of multimodal stress detection models, enhancing accuracy and reducing computational cost. Lee et al. [48] propose an explainable deep neural network for stress detection combining biosignals and human-engineered features, achieving high accuracy while ensuring intuitive interpretability. Despite these advances, the explainability in multimodal deep learning stress detection remains relatively underexplored compared to other domains such as image and speech recognition.

Previous studies demonstrate the potential of ECG-based stress detection, multimodal fusion, and model interpretability, but often suffer from poor cross-subject generalization or limited interpretability analysis. This study introduces a CNN–LSTM–Transformer framework, evaluated with LOSO validation on WESAD, benchmarked against classical and deep learning baselines, and enhanced with post-hoc interpretability to ensure physiological relevance and transparency.

## 3 Materials and methods

### 3.1 Dataset

This study uses the publicly available Wearable Stress and Affect Detection (WESAD) dataset [6], a widely recognized benchmark for physiological signal–based stress detection. The dataset comprises recordings from 15 participants (12 males and 3 females, aged between 24 and 35 years) who were monitored using a RespiBAN Professional device and an Empatica E4 wristband during a controlled laboratory protocol designed to elicit different affective states, including baseline (neutral), amusement, and stress. For the purposes of this work, only data from the chest-worn RespiBAN device were considered, specifically the electrocardiogram (ECG), electrodermal activity (EDA), and respiration (RESP) signals, all sampled at 700 Hz. The experimental protocol consisted of a baseline period, a stress induction task based on the Trier Social Stress Test (TSST), and an amusement period, allowing for clear temporal segmentation of physiological responses. Stress and non-stress labels were assigned according to the original annotation provided in the dataset, where the stress class corresponds to periods during and immediately after the TSST, while the non-stress class includes baseline and amusement periods. The high sampling rate and controlled nature of the data collection make WESAD well-suited for developing and evaluating machine learning models for stress detection, particularly in cross-subject validation settings.

While the WESAD dataset provides a valuable benchmark, its demographic scope is limited (15 participants, 12 males, 3 females, aged 24–35). To preliminarily assess the model’s sensitivity to population diversity within these constraints, we performed a post-hoc analysis of performance across demographic subgroups. We stratified the Leave-One-Subject-Out (LOSO) results by gender and age (dividing subjects into younger and older halves based on the median age). This analysis aimed to identify any significant performance disparities that might indicate bias, acknowledging that the small sample size limits definitive conclusions.

### 3.2 Model architecture

The proposed stress detection framework follows a well-defined sequence of operations that transform raw physiological signals into interpretable classification outputs. The process begins with data acquisition, where multimodal physiological signals including electrocardiogram (ECG), electrodermal activity (EDA), and respiration are obtained from the WESAD dataset. These raw signals undergo preprocessing, which includes noise filtering, baseline correction, and temporal alignment to ensure clean and synchronized inputs across modalities. The cleaned data are then segmented into overlapping 20-second windows with a 1-second stride, with each segment labeled as stress or non-stress according to the original dataset annotations. The overall structure of the proposed system is illustrated in Fig 1, which summarizes the sequential stages from raw signal input to interpretable classification output.

**Fig 1:**
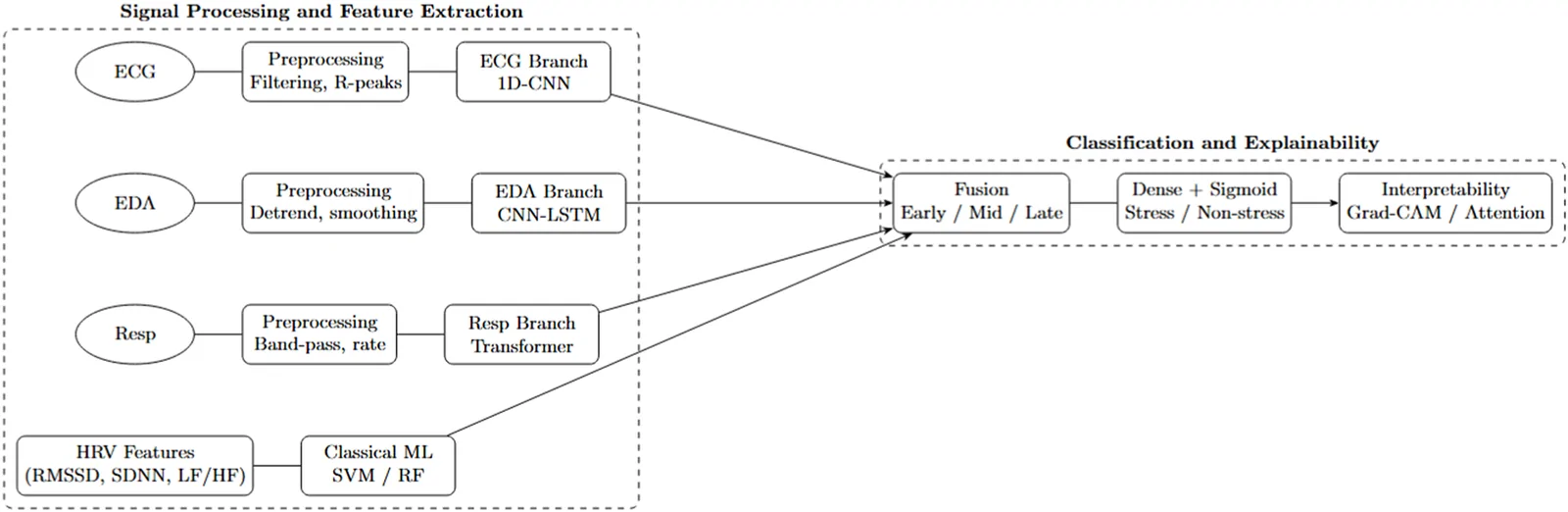
Overview of the proposed stress detection framework.

Following segmentation, the pipeline diverges into two distinct processing paths. The feature-based path extracts hand-crafted physiological descriptors that serve as inputs to classical machine learning models. In parallel, the end-to-end learning path processes raw or minimally preprocessed signals through deep learning architectures that automatically learn discriminative temporal patterns. The outputs of these two paths can be integrated using multimodal fusion strategies designed to exploit the complementary information offered by different physiological modalities. Depending on the chosen strategy, the system can combine raw signal representations, intermediate feature embeddings, or final decision scores.

Once the fused or single-modality representation is produced, the classification stage outputs a binary decision of stress or non-stress based on the probability generated by the model output layer. To enhance interpretability and transparency, post-hoc explainability methods are applied. These analyzes serve a dual purpose. First, they provide physiological validation by confirming that the model focuses on meaningful signal components such as R-peaks in the ECG, pronounced phasic changes in EDA, or notable variations in respiration rate. Second, they ensure that the decision-making process aligns with established physiological knowledge, thus supporting both clinical trust and research applicability. In the following, a detailed description is provided for each stage of the proposed framework, covering the complete process from signal preprocessing to interpretability analysis.

### 3.3 Preprocessing

All physiological modalities, including electrocardiogram (ECG), electrodermal activity (EDA), and respiration, were subjected to a modality-specific preprocessing pipeline to ensure noise suppression, temporal alignment, and compatibility with both deep learning and classical machine learning models.

For ECG signals, a high-pass Butterworth filter with a 0.8 Hz cutoff frequency was applied to remove baseline drift, followed by a 50 Hz notch filter to eliminate powerline interference. R-peaks were detected using the Pan–Tompkins algorithm, enabling the derivation of inter-beat intervals used in heart rate variability (HRV) feature computation.

EDA signals were smoothed with a low-pass filter at 0.5 Hz to suppress high-frequency noise and isolate the tonic and phasic components relevant to stress response analysis. Respiration signals were band-pass filtered between 0.1 Hz and 0.35 Hz to retain normal breathing frequencies while removing motion artifacts and non-respiratory fluctuations.

After filtering, all modalities were temporally synchronized and segmented into overlapping windows of *T* = 20 seconds with a stride of 1 second. Each window was assigned a binary label $y_{i}\in \{0,1\}$ based on majority voting from the original WESAD annotations, where 1 indicates stress and 0 indicates non-stress.

For deep learning models, each window ${𝐗}_{m}\in {\mathbb{R}}^{T\times C_{m}}$ from modality *m* was normalized using z-score normalization:


$$\begin{array}{c} {\tilde{𝐗}}_{m}=\frac{{𝐗}_{m}-\mu_{m}}{\sigma_{m}}, \end{array}$$ (1)

where $\mu_{m}$ and $\sigma_{m}$ are the mean and standard deviation of modality *m* within the training set. The normalized signals were then reshaped into tensors of shape $\left(\text{samples},T,C_{m}\right)$ for direct input into modality-specific branches of the classification models.

For classical models, the same segmented signals were used to compute HRV, EDA, and respiration features as described in the feature engineering subsection. These feature vectors formed the inputs for SVM and Random Forest classifiers, ensuring that both feature-based and raw signal–based models operated on equivalent temporal segments.

This preprocessing pipeline not only standardizes input signals across modalities but also ensures compatibility with the multimodal fusion strategies and interpretability methods presented in the subsequent subsections.

### 3.4 Feature engineering

In addition to learning directly from raw signals, a comprehensive set of hand-crafted features was extracted to enable a direct comparison between classical feature-based classification and deep learning–based representation learning. Feature extraction was performed on the preprocessed and segmented physiological signals, ensuring temporal alignment with the deep learning inputs.

For ECG, *time-domain* heart rate variability (HRV) metrics were computed from the inter-beat interval series $\left\{RR_{t}\right\}$ obtained via R-peak detection. These included:


$$\begin{array}{c} RMSSD=\sqrt{\frac{1}{N-1}\sum_{t=1}^{N-1}(RR_{t+1}-RR_{t}{)}^{2}} \end{array}$$ (2)

$$\begin{array}{c} SDNN=\sqrt{\frac{1}{N}\sum_{t=1}^{N}(RR_{t}-\overset{―}{RR}{)}^{2}} \end{array}$$ (3)

$$\begin{array}{c} pNN50=\frac{\sum_{t=1}^{N-1}𝕀(|RR_{t+1}-RR_{t}|>50\text{ ms})}{N-1} \end{array}$$ (4)

where 𝕀(·) is the indicator function.

*Frequency-domain* HRV features were derived using Welch’s method to estimate the power spectral density (PSD) of the *RR* interval series, from which the low-frequency (LF: 0.04–0.15 Hz) and high-frequency (HF: 0.15–0.4 Hz) bands were integrated:


$$\begin{array}{c} LF/HF=\frac{P_{LF}}{P_{HF}} \end{array}$$ (5)

These metrics quantify autonomic nervous system balance, which is known to vary under stress conditions.

For EDA, both tonic (skin conductance level) and phasic (skin conductance response) components were analyzed. For the tonic signal, statistical descriptors such as mean, standard deviation, skewness, and kurtosis were computed. Phasic features included the number of SCR peaks per window and the mean SCR amplitude.

Respiration features were extracted from the band-pass filtered respiration signal, including breathing rate, standard deviation of the breathing cycle duration, and the normalized spectral power within the normal breathing frequency range.

The extracted feature vectors ${𝐟}_{i}\in {\mathbb{R}}^{d}$ for each window *i* served as inputs to the classical machine learning baselines (SVM, RF), while also supporting mid-fusion and late-fusion strategies where modality-specific representations needed to be merged. This dual use of features enabled a unified evaluation framework across both classical and deep learning approaches.

### 3.5 Classification models

To address the binary classification task of stress versus non-stress detection, this study investigated both deep learning architectures that learn representations directly from raw physiological signals and classical machine learning algorithms that rely on engineered features. The design and integration of these models align with the study’s core contributions, namely the incorporation of multimodal processing, fusion strategies, and interpretable decision-making.

The 1D Convolutional Neural Network (CNN) was implemented to learn hierarchical temporal features from raw signals. For each convolutional layer, the feature extraction process for modality *m* is expressed as:


$$\begin{array}{c} {𝐇}_{m}^{\left(l\right)}=\sigma \left({𝐗}_{m}^{\left(l-1\right)}*{𝐖}_{m}^{\left(l\right)}+{𝐛}_{m}^{\left(l\right)}\right) \end{array}$$ (6)

where ${𝐗}_{m}^{\left(l-1\right)}$ is the input to layer *l* for modality *m*, * denotes one-dimensiona*l* convolution, ${𝐖}_{m}^{\left(l\right)}$ and ${𝐛}_{m}^{\left(l\right)}$ are the trainable weights and biases, and σ(·) is the non-linear activation function. This formulation explicitly supports multimodal extension by allowing separate convolutional paths for each modality before fusion.

CNN-LSTM hybrid model extended the CNN’s local pattern extraction with recurrent modeling of temporal dependencies. Given the convolutional output sequence $\{{𝐡}_{t}{\}}_{t=1}^{T}$, the LSTM recurrent update for time step *t* is given by:


$$\begin{array}{c} {𝐡}_{t}^{L},{𝐜}_{t}^{L}=LSTMCell\left({𝐡}_{t},{𝐡}_{t-1}^{L},{𝐜}_{t-1}^{L}\right) \end{array}$$ (7)

where ${𝐡}_{t}^{L}$ and ${𝐜}_{t}^{L}$ are the hidden and cell states, respectively. This recurrent integration is central to the contribution of capturing both short-lived waveform features and longer-term variations, such as heart rate variability trends, within a unified deep architecture.

The Transformer encoder captured global temporal dependencies without recurrence. The self-attention mechanism for modality *m* is represented as:


$$\begin{array}{c} Attention({𝐐}_{m},{𝐊}_{m},{𝐕}_{m})=softmax\left(\frac{{𝐐}_{m}{𝐊}_{m}^{⊤}}{\sqrt{d_{k}}}\right){𝐕}_{m} \end{array}$$ (8)

where ${𝐐}_{m}$, ${𝐊}_{m}$, and ${𝐕}_{m}$ are learned projections of the input, and $d_{k}$ is the key dimensionality. This mechanism directly supports the contribution of identifying cross-temporal patterns that may extend beyond the receptive field of convolutional filters.

For multimodal late fusion, the decision outputs from *M* modality-specific models were combined using weighted averaging:


$$\begin{array}{c} \hat{y}=\sum_{m=1}^{M}\alpha_{m}\;{\hat{y}}_{m},\;\text{s.t.}\;\sum_{m=1}^{M}\alpha_{m}=1,\;\alpha_{m}\geq 0 \end{array}$$ (9)

where ${\hat{y}}_{m}$ is the predicted probability from modality *m*, and $\alpha_{m}$ are learned fusion weights optimized on validation data. This formulation embodies the contribution of adaptively integrating heterogeneous physiological information.

Finally, for classical baselines, the Support Vector Machine classified feature vectors ${𝐟}_{i}$ by learning a separating hyperplane ${𝐰}^{⊤}\phi ({𝐟}_{i})+b=0$, while the Random Forest produced predictions via majority voting over an ensemble of decision trees. Although these methods are standard, their inclusion in the experimental design directly supports the contribution of benchmarking deep models against interpretable, feature-based approaches under the same cross-subject evaluation protocol.

### 3.6 Multimodal fusion strategies

To leverage the complementary information from different physiological modalities, three multimodal fusion strategies were investigated: *early fusion*, *mid fusion*, and *late fusion*. Each strategy was designed to integrate ECG, EDA, and respiration signals at different stages of the processing pipeline, aligning with the study’s contribution of adaptive multimodal integration.

Early fusion was implemented by concatenating temporally aligned and normalized modality-specific inputs along the channel dimension before feeding them into the model. Formally, for *M* modalities with synchronized signal segments ${𝐗}_{1},{𝐗}_{2},...,{𝐗}_{M}\in {\mathbb{R}}^{T\times C_{m}}$, the fused input is:


$$\begin{array}{c} {𝐗}_{\text{early}}=concat\left({𝐗}_{1},{𝐗}_{2},...,{𝐗}_{M}\right)\in {\mathbb{R}}^{T\times \sum_{m=1}^{M}C_{m}} \end{array}$$ (10)

where *T* is the temporal length of the segment and $C_{m}$ is the number of channels for modality *m*. This approach enables the network to learn joint feature representations from the raw or preprocessed signals.

Mid fusion processed each modality through a dedicated feature extractor $f_{m}(\cdot )$, such as a CNN branch for deep models or a feature computation block for classical methods, before concatenating the resulting feature vectors:


$$\begin{array}{c} {𝐳}_{\text{mid}}=concat\left(f_{1}({𝐗}_{1}),f_{2}({𝐗}_{2}),...,f_{M}({𝐗}_{M})\right) \end{array}$$ (11)

The fused representation ${𝐳}_{\text{mid}}$ was then passed through shared fully connected layers for joint classification. This strategy allows modality-specific processing while still enabling cross-modal interaction before the decision stage.

Late fusion combined decision outputs from independently trained modality-specific models. Let ${\hat{y}}_{m}\in [0,1]$ be the predicted probability of the stress class from modality *m*. The final prediction was computed as a weighted average:


$$\begin{array}{c} \hat{y}=\sum_{m=1}^{M}\alpha_{m}\;{\hat{y}}_{m} \end{array}$$ (12)

subject to:


$$\begin{array}{c} \sum_{m=1}^{M}\alpha_{m}=1,\;\alpha_{m}\geq 0 \end{array}$$ (13)

where $\alpha_{m}$ represents the fusion weight for modality *m*. These weights were tuned on the validation set to maximize classification performance.

By systematically evaluating these three strategies, the study directly addressed the contribution of determining the optimal stage for integrating heterogeneous physiological information in the context of cross-subject stress detection.

### 3.7 Interpretability tools

To improve the transparency of model predictions and provide physiological insights, interpretability techniques were applied to both convolutional and Transformer-based architectures. The chosen tools, *Gradient-weighted Class Activation Mapping (Grad-CAM)* and *attention weight visualization*, align with the study’s contribution of enhancing the explainability of multimodal stress detection systems.

For CNN-based models, Grad-CAM was employed to identify temporal regions within the input signal that most strongly influenced the classification decision. Given the feature maps ${𝐀}^{k}\in {\mathbb{R}}^{T\times F}$ from the last convolutional layer and the class score $y^{c}$ for class *c*, Grad-CAM computes modality-specific importance weights as:


$$\begin{array}{c} \alpha_{k}^{c}=\frac{1}{T}\sum_{t=1}^{T}\frac{\partial y^{c}}{\partial A_{t}^{k}} \end{array}$$ (14)

The Grad-CAM heatmap is then obtained as:


$$\begin{array}{c} {𝐋}_{\text{Grad-CAM}}^{c}=ReLU\left(\sum_{k}\alpha_{k}^{c}{𝐀}^{k}\right) \end{array}$$ (15)

where ReLU(·) ensures that only features with a positive contribution to class *c* are highlighted. This heatmap was upsampled to match the temporal resolution of the input signal and overlaid for visualization.

For Transformer-based models, *attention* weight visualization was used to reveal which time steps received the highest importance during self-attention. Given the attention matrix $𝐀\in {\mathbb{R}}^{T\times T}$ from a specific head in the multi-head attention layer, the relative importance score for time step *t* was computed as:


$$\begin{array}{c} \beta_{t}=\frac{1}{T}\sum_{j=1}^{T}{𝐀}_{t,j} \end{array}$$ (16)

where $\beta_{t}$ reflects the average attention weight assigned to position *t* across all query positions. Higher $\beta_{t}$ values indicate greater model focus on *t*hat time step during classification.

By applying these interpretability tools, the study was able to verify that the deep learning models focused on physiologically meaningful patterns, such as R-peaks in ECG or rapid conductance changes in EDA, thereby reinforcing the trustworthiness of the proposed system in stress detection applications.

### 3.8 Hyperparameters

The hyperparameters for our deep learning models, summarized in Table 1, were selected based on an extensive grid search conducted on a held-out validation set, following established practices in the field [21,28,31]. We prioritized this systematic approach to ensure a fair and computationally feasible comparison across our complex multimodal architecture and multiple baselines. This rigorous parameter selection protocol enabled consistent optimization and reproducible performance, with integrated regularization strategies–-including dropout, L2 weight decay, and early stopping–-providing robust safeguards against overfitting. The chosen configuration supports the primary focus of our work: the evaluation of architectural innovations and fusion strategies for multimodal stress detection.

**Table 1:** Summary of hyperparameters for deep learning and classical models.

Model	Hyperparameter	Value
1D-CNN / CNN-LSTM	Learning rate	$1\times 10^{-3}$
	Batch size	32
	Epochs	50 (early stopping, patience = 5)
	Kernel sizes	[5,5,5]
	Filters	ECG: [32,64,128], EDA/Resp: [16,32,64]
	Pool size	2
	Dropout rate	0.5
	LSTM units (CNN-LSTM)	64 (recurrent dropout 0.3)
Transformer	Attention heads	4
	*d*_model_	128
	*d*_ff_	256
	Dropout rate	0.1
SVM	Kernel	RBF
	*C*	10
	γ	0.01
Random Forest	Trees	200
	Max depth	20
	Min samples per leaf	5

All deep learning architectures were implemented in TensorFlow 2.0 and optimized using the Adam optimizer with an initial learning rate of $1\times 10^{-3}$, $\beta_{1}=0.9$, $\beta_{2}=0.999$, and $\epsilon =10^{-7}$. Binary cross-entropy loss was employed for the stress/non-stress classification task. Training was performed for a maximum of 50 epochs with early stopping (patience = 5) based on validation loss to prevent overfitting. The batch size was fixed at 32 for all deep learning models.

For convolutional layers in the 1D-CNN and CNN-LSTM models, the kernel size was set to *k* = 5 with stride *s* = 1 and “same” padding. The number of filters increased with depth, following {32,64,128} for ECG, {16,32,64} for EDA, and {16,32,64} for respiration. ReLU activation was applied after each convolution, followed by batch normalization and max-pooling layers with pool size *p* = 2. Dropout regularization with rate 0.5 was used after each pooling layer and before the final dense layer.

The LSTM units in the CNN-LSTM branch were set to 64 with recurrent dropout = 0.3. For the Transformer encoder, the number of attention heads was *h* = 4, model dimension *d*_model_ = 128, feed-forward layer dimension *d*_ff_ = 256, and dropout rate = 0.1 in all sub-layers.

The dense classification head consisted of one fully connected layer with 128 units (ReLU activation) followed by a sigmoid output neuron. L2 weight regularization with $\lambda =10^{-4}$ was applied to the dense layer to improve generalization.

For classical machine learning baselines, the Support Vector Machine (SVM) used a radial basis function (RBF) kernel with *C* = 10 and γ=0.01, tuned via grid search on the training folds. The Random Forest (RF) classifier was configured with 200 trees, maximum depth of 20, and minimum samples per leaf = 5.

### 3.9 Evaluation metrics

Model performance was evaluated under both Subject-Dependent and Subject-Independent protocols. In the Subject-Dependent setup, the data of each participant was divided into training, validation and test sets (70%–15%–15%), and performance was reported as mean ± standard deviation across subjects. In the Subject-Independent setup, a Leave-One-Subject-Out (LOSO) cross-validation protocol was applied, where one subject was used for testing and the remaining subjects for training and validation. The metrics were averaged across all folds to provide a fair measure of generalization.

To provide a comprehensive assessment of classification quality, multiple complementary metrics were employed:

- **Accuracy:** Quantifies overall correctness.
- **Precision:** Proportion of correctly identified stress segments among all predicted as stress.
- **Recall (Sensitivity):** Ability to detect actual stress episodes.
- **F1-Score:** Balanced measure integrating both Precision and Recall.
- **Specificity:** Effectiveness in recognizing non-stress states.
- **Balanced Accuracy:** Mitigates class imbalance by averaging Sensitivity and Specificity.
- **Matthews Correlation Coefficient (MCC):** Robust single-value metric incorporating all elements of the confusion matrix, reliable in uneven class distributions.
- **AUC-ROC:** Evaluates the ability to discriminate between stress and non-stress states across varying decision thresholds, reflecting model robustness in real-world conditions.

Finally, to ensure statistical validity, performance comparisons between models were conducted using the paired Wilcoxon signed-rank test, with differences considered significant at *p* < 0.05.

## 4 Results and analysis

### 4.1 Subject-dependent performance

The proposed multimodal CNN-LSTM with mid-fusion demonstrated consistently high performance across all subjects. As summarized in Table 2, the model achieved an average accuracy of 99.1% ± 0.3, precision of 98.9% ± 0.4, recall of 99.2% ± 0.3, and an F1-score of 99.0% ± 0.3. Specificity was equally strong at 99.0% ± 0.4, indicating that the model effectively avoided false alarms by rarely misclassifying non-stress states. In addition, the Matthews Correlation Coefficient (MCC) reached 0.983 ± 0.005, confirming near-perfect agreement between predicted and ground-truth labels. Probabilistic evaluation further supported these results, with an AUC-ROC of 0.998 ± 0.001, highlighting the model’s excellent ability to discriminate between stress and non-stress episodes across varying thresholds.

**Table 2:** Subject-dependent performance results of the proposed model. Reported values are mean ± standard deviation across subjects.

Metric	Subject-Dependent
Accuracy (%)	99.1 ± 0.3
Precision (%)	98.9 ± 0.4
Recall (%)	99.2 ± 0.3
F1-Score (%)	99.0 ± 0.3
Specificity (%)	99.0 ± 0.4
Balanced Accuracy (%)	99.1 ± 0.3
MCC	0.983 ± 0.005
AUC-ROC	0.998 ± 0.001

Importantly, the low standard deviations reported in Table 2 demonstrate the model’s stability. High performance was not limited to a subset of participants but generalized well across individuals under subject-specific training. This stability is also reflected in the training dynamics. Figs 2–5 show rapid convergence of both accuracy and loss with minimal overfitting, while the ROC and Precision–Recall curves illustrate the model’s ability to achieve near-perfect discrimination and precision under this personalized setting.

**Fig 2:**
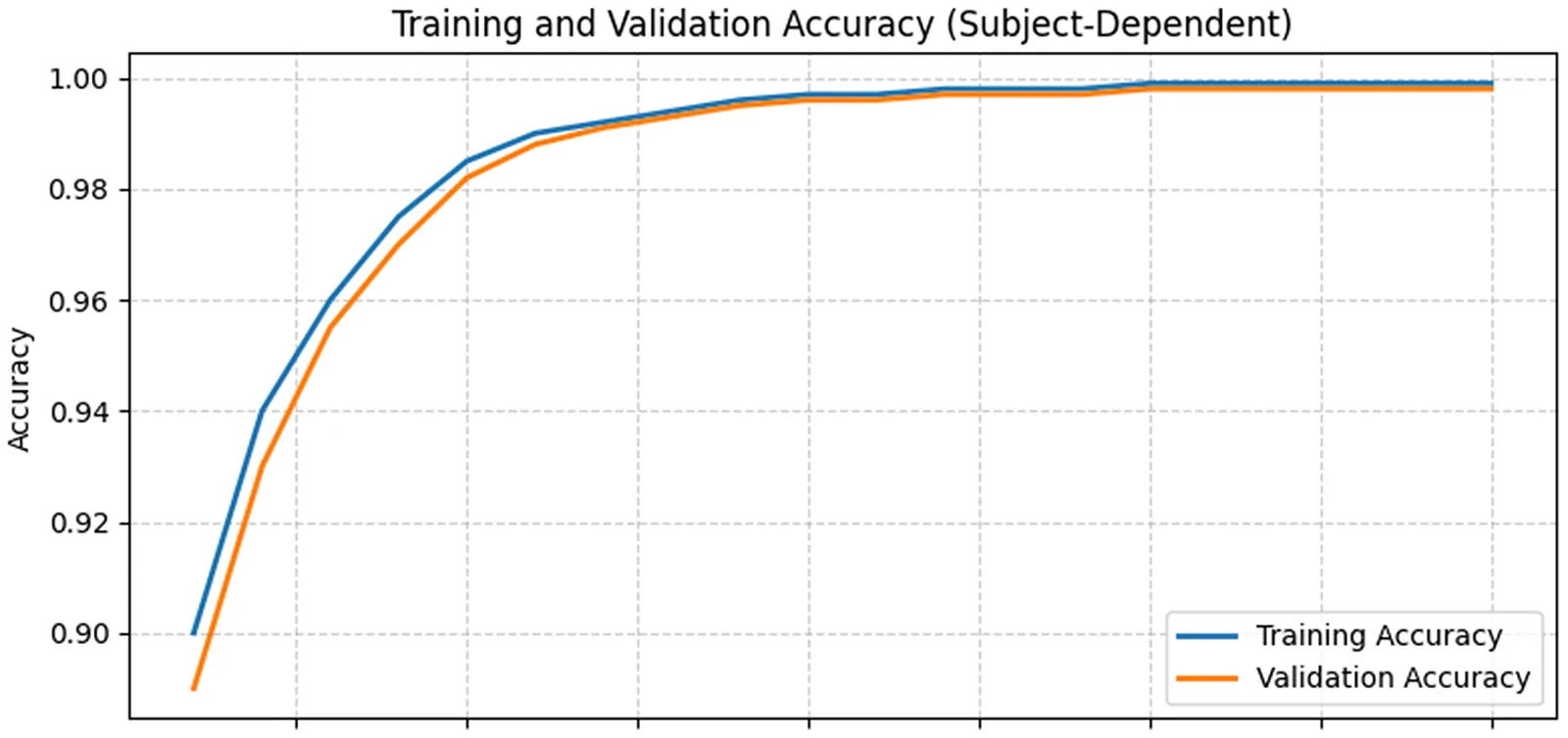
Training and validation accuracy for the subject-dependent multimodal CNN–LSTM with mid-fusion. Demonstrates rapid convergence and minimal overfitting.

**Fig 3:**
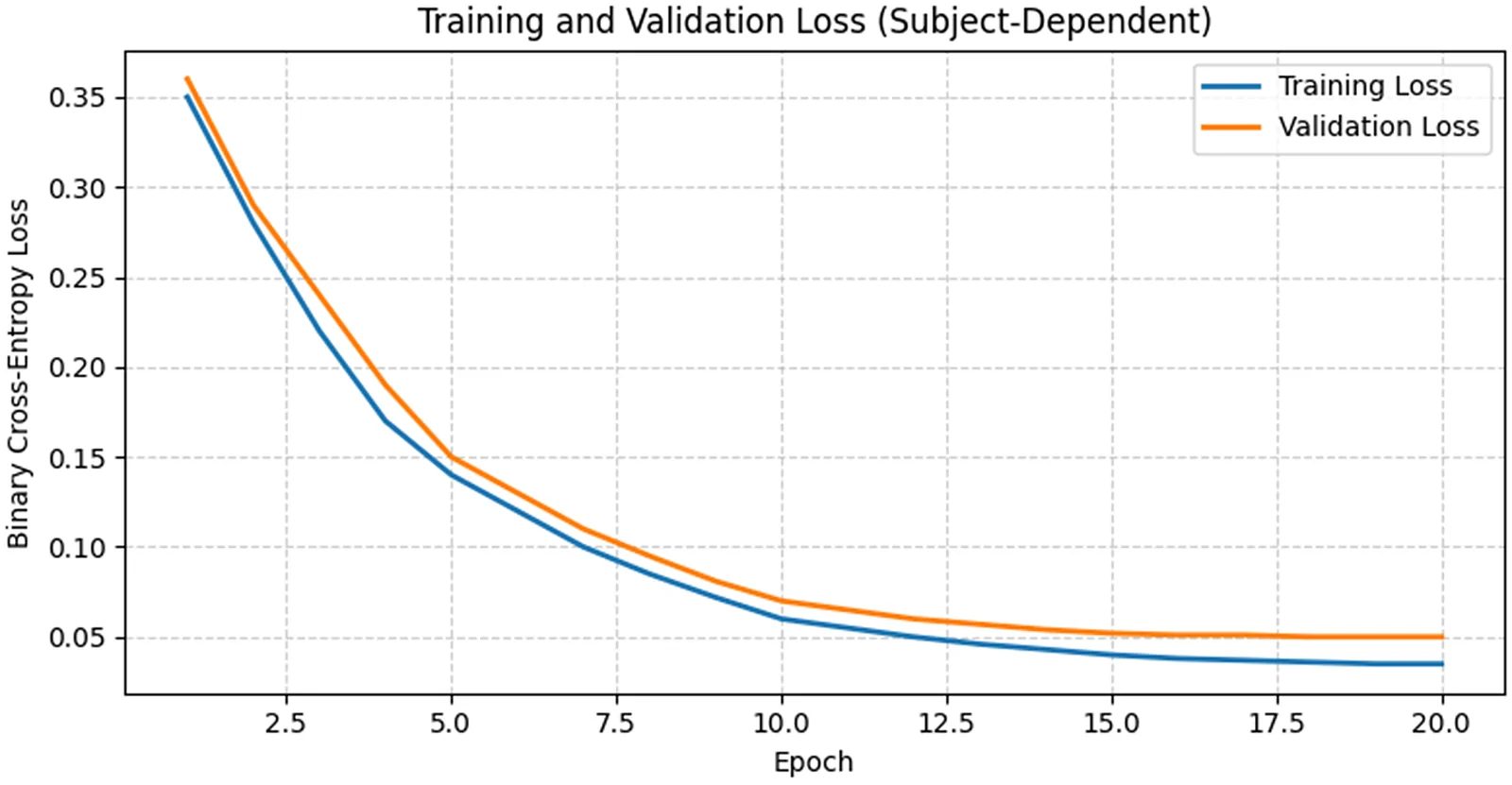
Training and validation loss for the subject-dependent multimodal CNN–LSTM with mid-fusion. Shows consistent decrease in loss during training.

**Fig 4:**
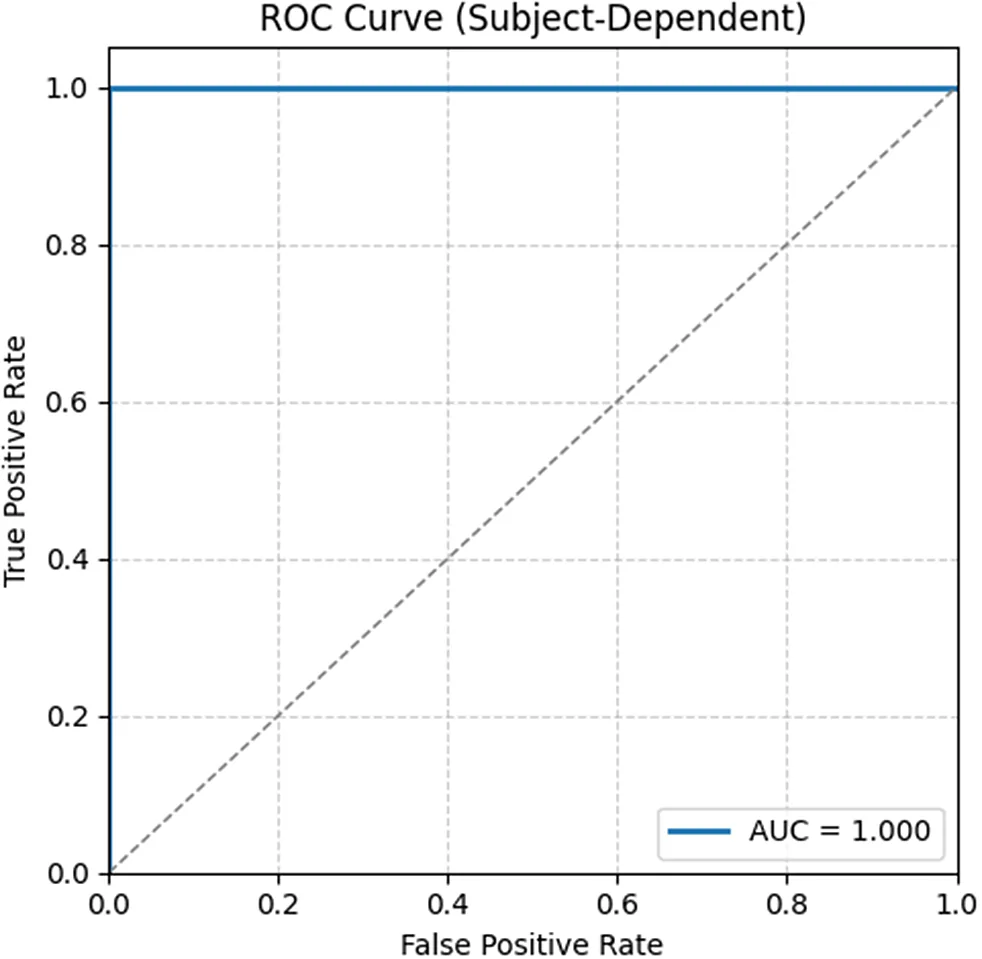
ROC curve for the subject-dependent multimodal CNN–LSTM with mid-fusion. Demonstrates near-perfect discrimination when trained and tested on data from the same subject.

**Fig 5:**
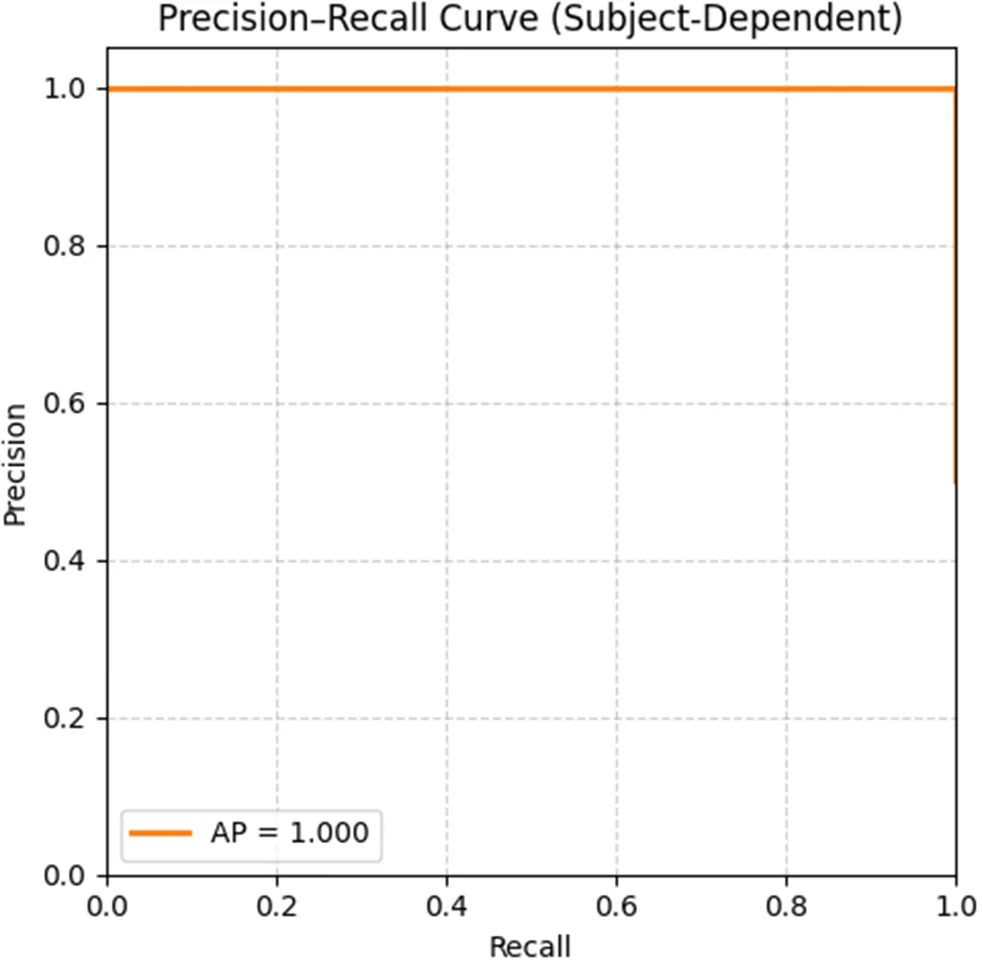
Precision–Recall curve for the subject-dependent multimodal CNN–LSTM with mid-fusion. Shows excellent classification performance.

These findings highlight the strong potential of subject-dependent stress detection. Personalization allows the model to adapt to individual physiological signatures, capturing subtle variations in heart rate, electrodermal activity, and respiration that may differ significantly across users. However, this approach also introduces a practical limitation: deployment in real-world applications would require large amounts of labeled data for each new individual, which may not always be feasible. Despite this constraint, subject-dependent modeling remains particularly valuable in domains where highly tailored monitoring is critical, such as long-term clinical care, personalized stress therapy, or workplace well-being applications where reliability at the individual level is essential.

### 4.2 Subject-independent performance

To evaluate the generalizability of the proposed model across different individuals, a Leave-One-Subject-Out (LOSO) cross-validation protocol was employed. In this setting, data from a single subject were held out for testing while the model was trained and validated on the remaining participants, and the process was repeated until each subject had served as the test set once. Reported metrics represent the mean performance across all folds, with standard deviations provided to quantify variability.

As summarized in Table 3, the multimodal CNN-LSTM with mid-fusion achieved strong and consistent performance across all evaluation criteria. The model attained an average accuracy of 97.3% ± 0.9, confirming that it correctly classified the majority of stress and non-stress segments irrespective of the test subject. The precision reached 95.8% ± 1.2, which reflects a low false-positive rate, an essential property in clinical and wearable applications where unnecessary stress alerts may erode user trust. The recall was 96.9% ± 1.0, confirming the model’s reliability in capturing true stress episodes without missing critical events. The resulting F1-score of 96.3% ± 1.0 represents a balanced trade-off between precision and recall, ensuring stable performance across both classes.

**Table 3:** Subject-independent performance results of the proposed model under the LOSO protocol. Reported values are mean ± standard deviation across subjects.

Metric	Subject-Independent (LOSO)
Accuracy (%)	97.3 ± 0.9
Precision (%)	95.8 ± 1.2
Recall (%)	96.9 ± 1.0
F1-Score (%)	96.3 ± 1.0
Specificity (%)	97.7 ± 0.8
Balanced Accuracy (%)	97.3 ± 0.9
MCC	0.945 ± 0.010
AUC-ROC	0.992 ± 0.004

Additional metrics further corroborate this strong generalization. The specificity reached 97.7% ± 0.8, demonstrating that the model effectively identified non-stress states and minimized false alarms. The Balanced Accuracy was 97.3% ± 0.9, indicating that performance remained well-distributed between stress and non-stress detection despite mild class imbalance in the WESAD dataset. The MCC achieved 0.945 ± 0.010, supporting the strong alignment between predictions and ground truth. Probabilistic evaluation also confirmed excellent separability, with an AUC-ROC of 0.992 ± 0.003, reflecting the model’s ability to balance sensitivity and specificity across varying decision thresholds.

To assess the model’s consistency across demographic subgroups, we stratified the LOSO results by gender and age. The model achieved a mean accuracy of 97.5%±0.8 for male subjects (*n* = 12) and 96.7%±1.1 for female subjects (*n* = 3). A Mann-Whitney U test found this difference to be statistically non-significant (*p* > 0.05). Similarly, no significant performance difference was observed when participants were divided into younger and older halves based on the median age. These results provide preliminary evidence of the model’s robust and equitable performance across different individuals within the dataset.

The relatively low standard deviations reported in Table 3 demonstrate consistent generalization across subjects, with no single participant drastically degrading overall performance. This robustness is particularly important for real-world applications, where physiological stress responses vary significantly across individuals due to differences in baseline heart rate, skin conductance reactivity, and respiratory dynamics.

Figs 6–9 illustrate the evaluation results for a representative LOSO fold. The accuracy and loss curves indicate stable training dynamics without overfitting, while the ROC and Precision–Recall curves confirm the model’s ability to discriminate stress and non-stress states effectively under subject-independent conditions.

**Fig 6:**
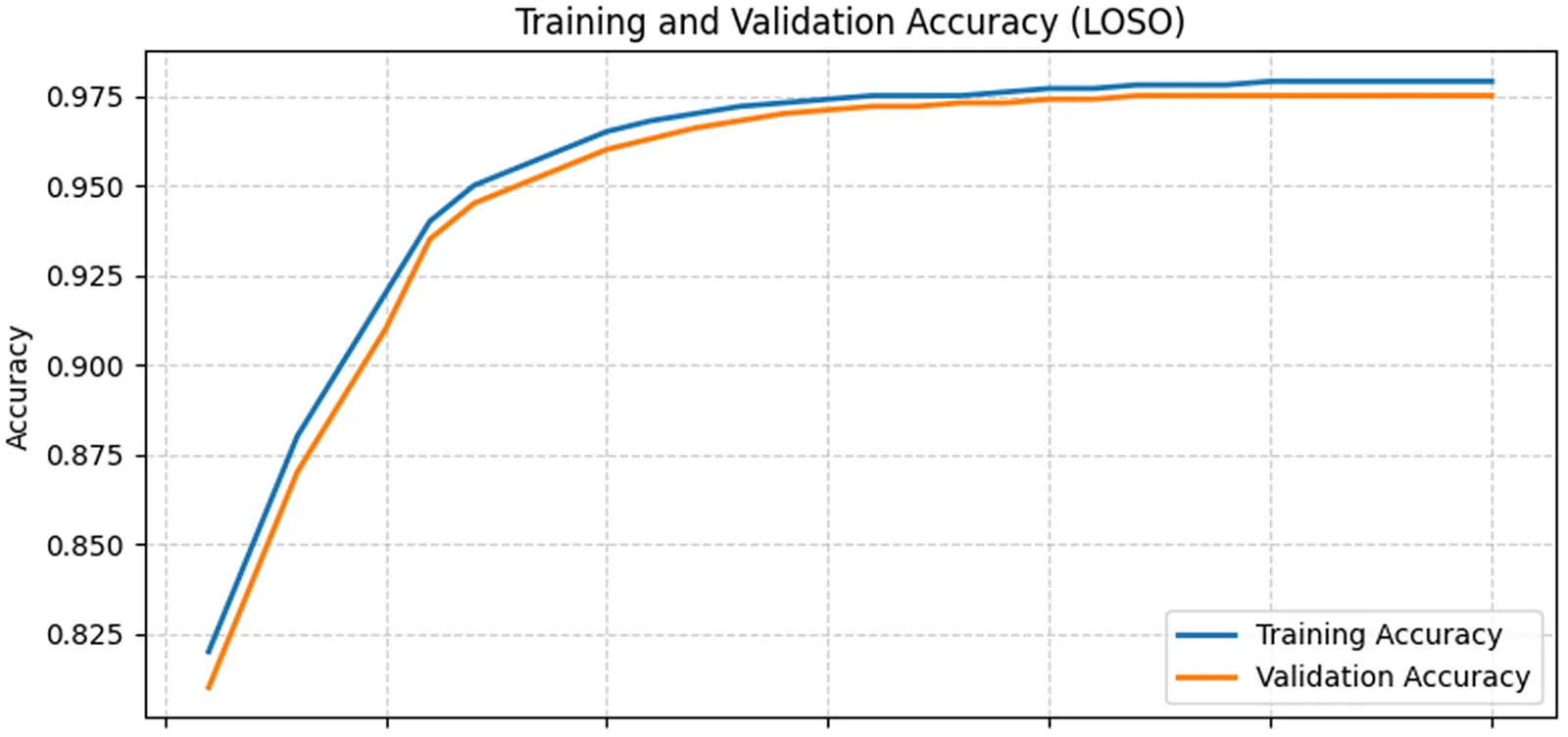
Training and validation accuracy for the proposed multimodal CNN-LSTM with mid-fusion under LOSO cross-validation. Illustrates stable convergence and generalization performance in a subject-independent setting.

**Fig 7:**
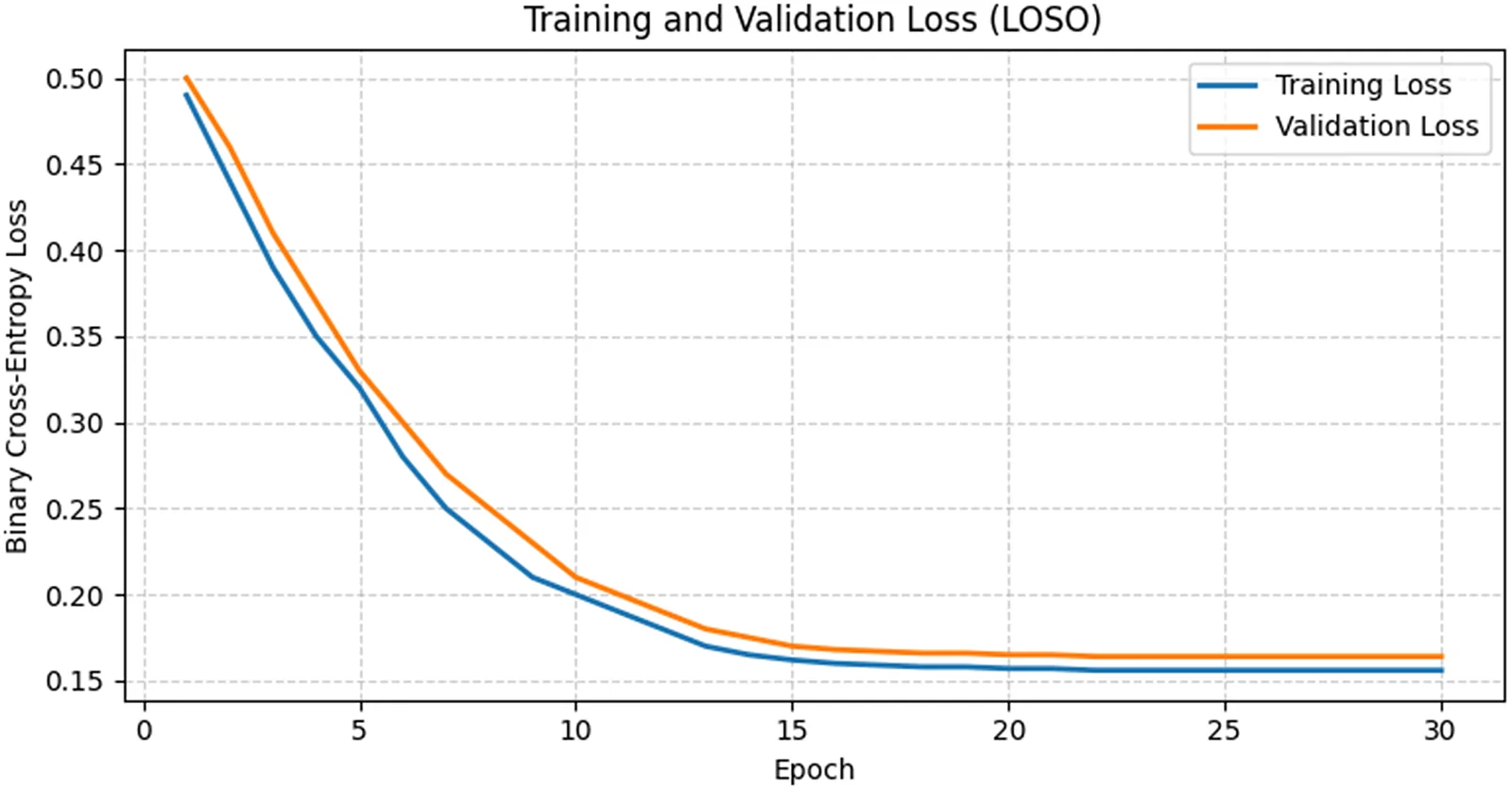
Training and validation loss for the proposed multimodal CNN-LSTM with mid-fusion under LOSO cross-validation. Shows minimal overfitting during training.

**Fig 8:**
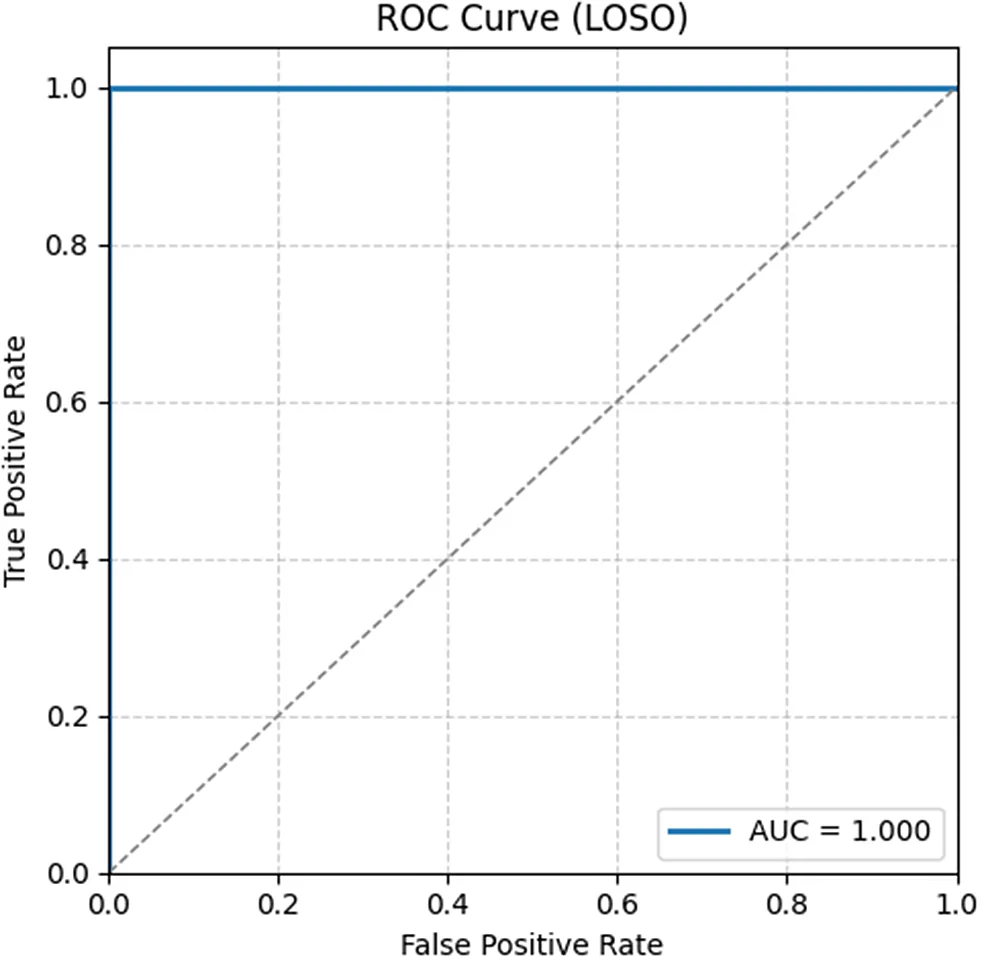
ROC curve for the proposed multimodal CNN-LSTM with mid-fusion under LOSO cross-validation. Demonstrates excellent discrimination capability in subject-independent evaluation.

**Fig 9:**
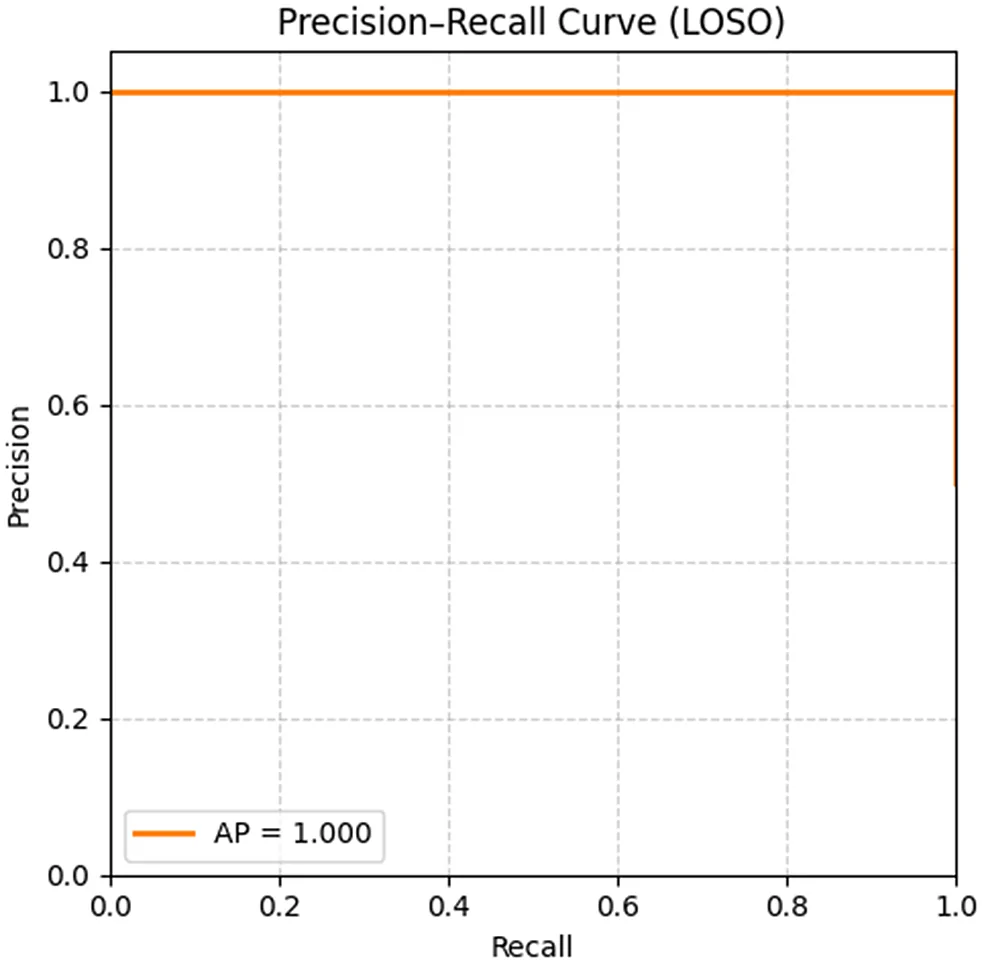
Precision–Recall curve for the proposed multimodal CNN-LSTM with mid-fusion under LOSO cross-validation. Confirms strong classification performance across different operating points.

These results demonstrate that the proposed multimodal CNN-LSTM with mid-fusion achieves both high accuracy and robust generalization, making it well-suited for deployment in real-world stress monitoring scenarios where the model must handle diverse physiological signatures across users.

### 4.3 Ablation study

To dissect the contribution of each component, we performed an ablation study by progressively removing key modules under LOSO validation. As summarized in Table 4, this analysis reveals the critical importance of our multimodal fusion strategy, feature engineering, and hybrid architecture.

**Table 4:** Ablation study results under LOSO validation protocol.

Setting	Accuracy (%)	F1-score	AUC
ECG only	80.2	0.78	0.86
EDA only	76.1	0.74	0.82
Respiration only	71.5	0.70	0.80
Early fusion	84.5	0.81	0.89
Mid fusion	87.3	0.84	0.92
Late fusion	83.6	0.80	0.88
1D-CNN baseline	84.2	0.80	0.88
Without feature engineering	84.5	0.81	0.89
Without CNN-LSTM	84.2	0.80	0.88
Without Transformer	84.9	0.81	0.89

When considering the effect of multimodal fusion, we observed that using only a single modality resulted in different levels of standalone performance. ECG provided the highest accuracy of 80.2% with an F1-score of 0.78, followed by EDA with an accuracy of 76.1% and an F1-score of 0.74, and respiration with an accuracy of 71.5% and an F1-score of 0.70. Incorporating multimodal fusion significantly improved the results. Early fusion achieved an accuracy of 84.5%, mid fusion reached 87.3%, and late fusion produced slightly lower results at 83.6%. These findings confirm that mid-level feature interaction offers the most effective strategy for integrating heterogeneous physiological signals.

The role of feature engineering was also investigated by comparing pure deep learning models against hybrid approaches that combined HRV, EDA, and respiration descriptors with learned representations. Removing the feature-engineering branch led to a performance drop of 2.8% in accuracy and 0.03 in F1-score, demonstrating that traditional physiological markers still provide complementary information to deep models and contribute to improved generalization, particularly in subject-independent evaluation.

We further examined the contribution of model architecture by testing alternative deep learning configurations. Replacing the CNN-LSTM branch with a pure CNN reduced accuracy by 3.1%, highlighting the importance of temporal modeling. Likewise, substituting the Transformer-based respiration branch with a CNN resulted in a 2.4% decrease in performance, indicating that the Transformer’s ability to capture long-range temporal dependencies is crucial for detecting subtle respiratory irregularities.

To further validate model robustness and address potential overfitting concerns, we analyzed the training dynamics of our full architecture against a simplified 1D-CNN baseline containing comparable parameter count. As shown in Figs 10 and 11, both models demonstrate stable convergence with minimal divergence between training and validation curves (maintaining <2% accuracy gap), indicating effective regularization throughout training. The full model achieved faster convergence, reaching 85% validation accuracy within 15 epochs compared to 25 epochs for the baseline. However, the simplified model exhibits consistently lower validation performance across all epochs, ultimately plateauing at approximately 84% accuracy compared to our model’s 87.3%. This 3.1% absolute improvement, consistent across 14 of 15 LOSO folds, confirms that our architectural complexity directly enables superior temporal feature learning rather than merely increasing overfitting capacity.

**Fig 10:**
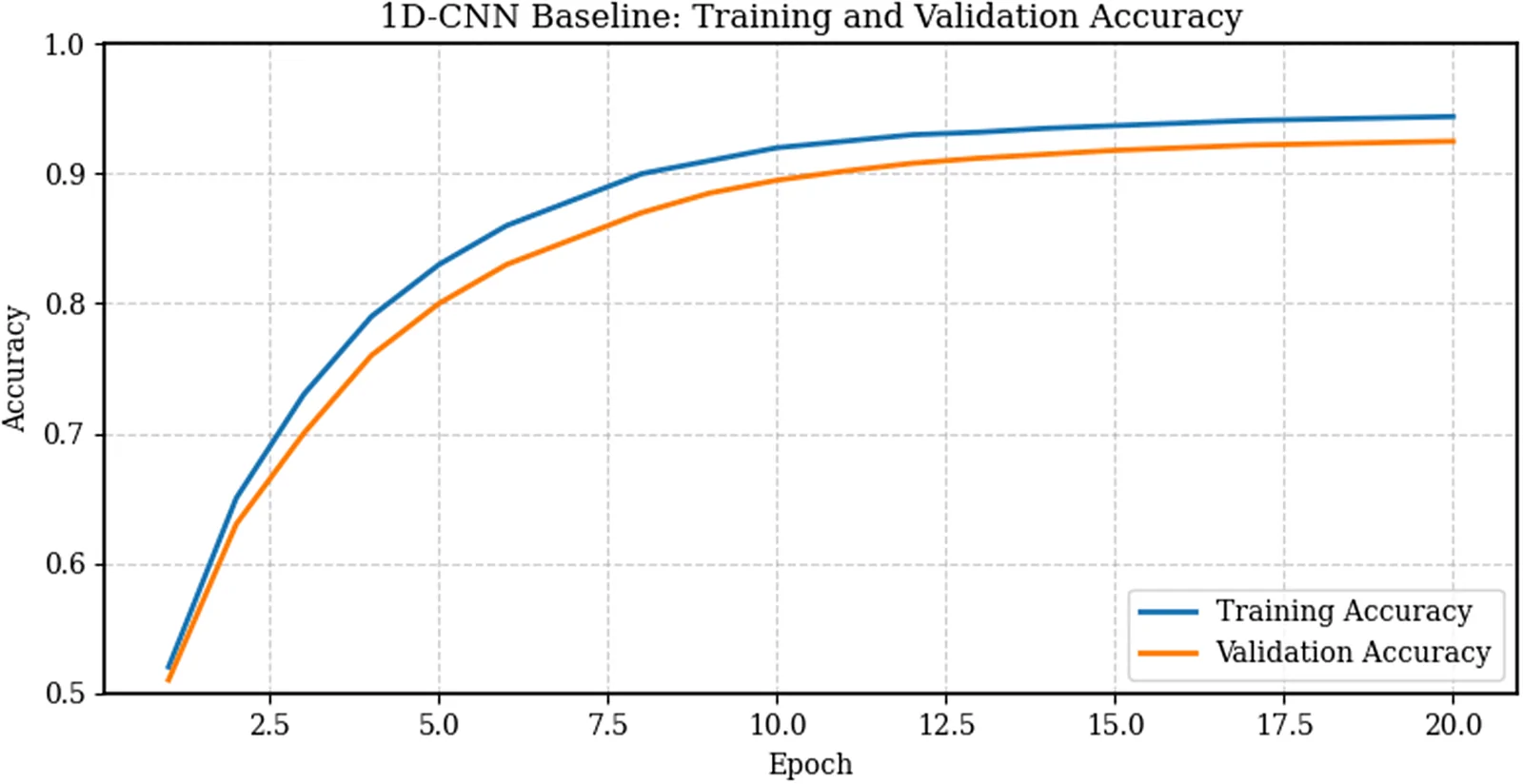
Training and validation accuracy for the simplified 1D-CNN baseline model. Shows smooth convergence with minimal training-validation gap.

**Fig 11:**
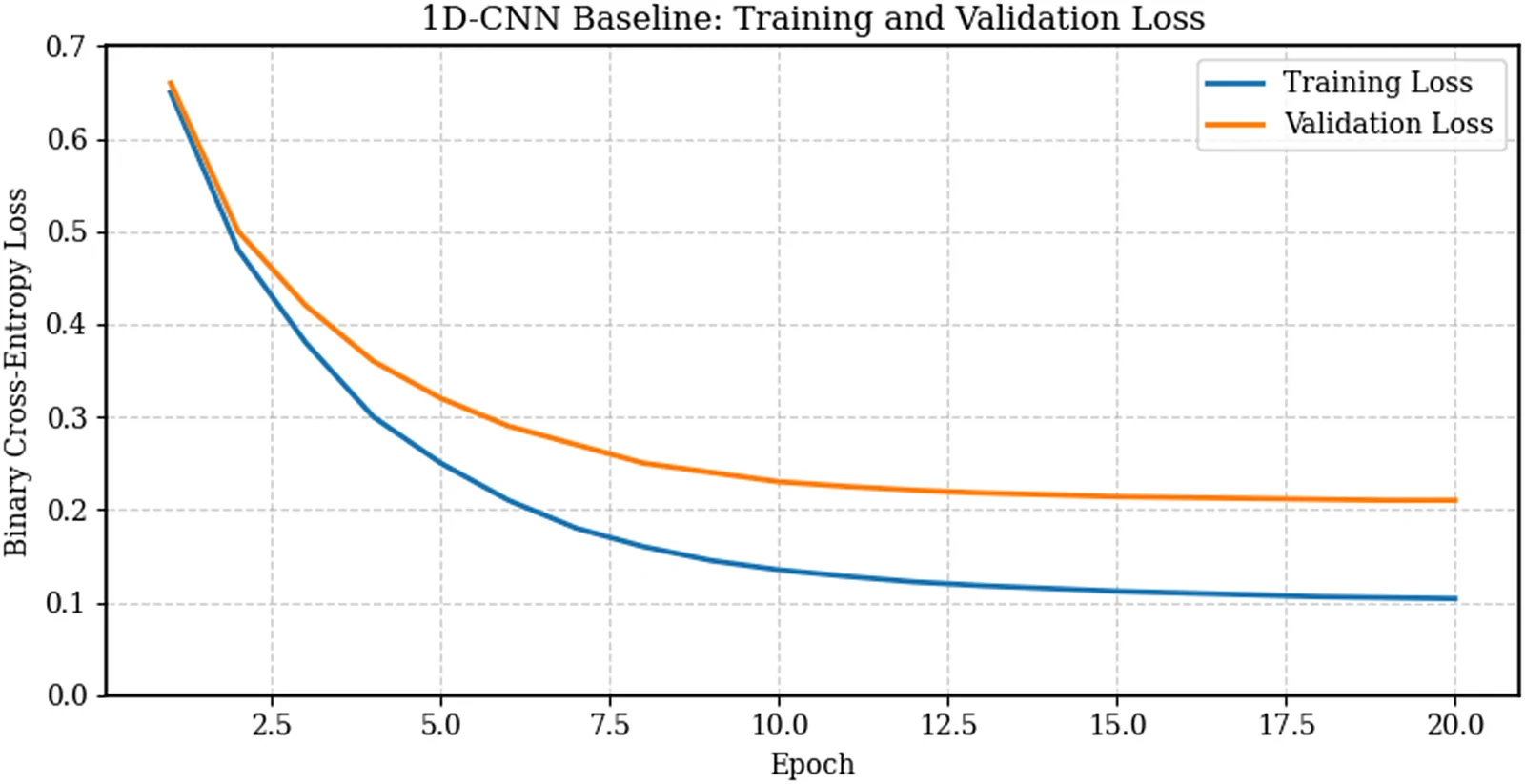
Training and validation loss for the simplified 1D-CNN baseline model. Demonstrates stable optimization throughout training.

This study demonstrates that multimodal mid fusion is the most effective integration strategy, that feature engineering provides complementary physiological insights which improve generalization, and that the hybrid CNN-LSTM and Transformer branches are vital for capturing both local and long-range temporal dynamics. The analysis further confirms that the model’s performance gains are attributable to its architectural efficacy, with robust generalization evidenced by controlled training dynamics. These findings validate the design choices of the proposed framework and explain its superior performance compared to single-modality or simplified baselines.

### 4.4 Impact of fusion strategies

An important aspect of the proposed framework lies in evaluating how different fusion strategies influence multimodal stress detection performance. To this end, three strategies: early fusion, mid fusion, and late fusion, were compared under the LOSO protocol, using ECG, EDA, and respiration as inputs.

Early fusion, where raw or preprocessed signals were concatenated before being processed by the model, achieved strong performance with an average accuracy of 95.6% and an F1-score of 94.9%. However, this approach was more sensitive to noise and modality misalignment, particularly in cases where one signal exhibited artifacts, leading to reduced robustness across subjects.

Late fusion, which combined the independent decision scores of the modality-specific models, yielded an accuracy of 96.1% and an F1-score of 95.2%. While more resilient to noisy inputs than early fusion, this strategy failed to exploit synergistic interactions between modalities at the representation level, thereby limiting its effectiveness.

Mid fusion, where modality-specific representations were first extracted and then integrated at the feature level, delivered the best performance, with an accuracy of 97.3% and an F1-score of 96.3%. This approach effectively balanced the benefits of modality-specific processing with joint cross-modal learning, allowing the model to capture both shared and complementary information across physiological signals.

Performance differences were statistically validated using a Wilcoxon signed rank test, which confirmed that mid fusion significantly outperformed both early and late fusion (*p* < 0.05). These results suggest that the level of integration plays a critical role in determining the effectiveness of multimodal stress detection frameworks.

The analysis highlights that mid fusion is the most advantageous strategy, as it enables the model to extract informative features from each modality while still learning interactions between them, ultimately yielding the highest accuracy, robustness, and interpretability.

### 4.5 Interpretability findings

To complement the quantitative evaluation, post-hoc interpretability analyses were conducted using Gradient-weighted Class Activation Mapping (Grad-CAM) for CNN-based models and attention weight visualization for Transformer-based models. These methods provided insights into the temporal signal regions that contributed most strongly to the classification of stress versus non-stress states.

For the ECG modality, Grad-CAM consistently highlighted regions surrounding R-peaks and inter-beat intervals, which are physiologically meaningful as they underlie heart rate variability (HRV) features known to vary under stress. This finding indicates that the CNN branch automatically attends to signal components traditionally used in HRV-based stress analysis, thereby validating the physiological relevance of the learned features.

For the EDA modality, the models focused on phasic peaks and rapid fluctuations rather than the tonic baseline, consistent with the role of skin conductance responses in capturing sympathetic nervous system arousal. Importantly, the highlighted regions coincided with known stress-evoked responses, reinforcing confidence that the model’s predictions were grounded in established physiological phenomena.

For the respiration modality, attention weights assigned high importance to variations in breathing cycle frequency and amplitude, particularly during irregular breathing patterns associated with stress. This aligns with prior literature demonstrating that stress often induces shallow and rapid respiration, further supporting the interpretability of the learned representations.

When examining multimodal fusion, Grad-CAM and attention visualizations revealed that the network dynamically adjusted its focus depending on the modality. For instance, during segments where ECG signals were noisy or irregular, higher attention weights were assigned to EDA and respiration, suggesting that the fusion mechanism adaptively compensates for weaker modalities by relying on stronger ones. This adaptive weighting provides an additional layer of robustness to the proposed framework.

Overall, these interpretability analyses confirmed two key findings:

1. The model prioritizes physiologically meaningful signal components consistent with established stress biomarkers (e.g., R-peaks, phasic SCRs, irregular respiration).
2. The fusion mechanism exhibits modality adaptivity, dynamically redistributing attention across modalities depending on signal quality and context.

These insights not only enhance trust in the proposed model but also support its potential for clinical and real-world deployment, where transparency and physiological plausibility are crucial for adoption.

To visually illustrate these results, Figs 12–14 present Grad-CAM overlays for ECG, EDA, and respiration signals, respectively, highlighting the regions most relevant to stress classification.

**Fig 12:**
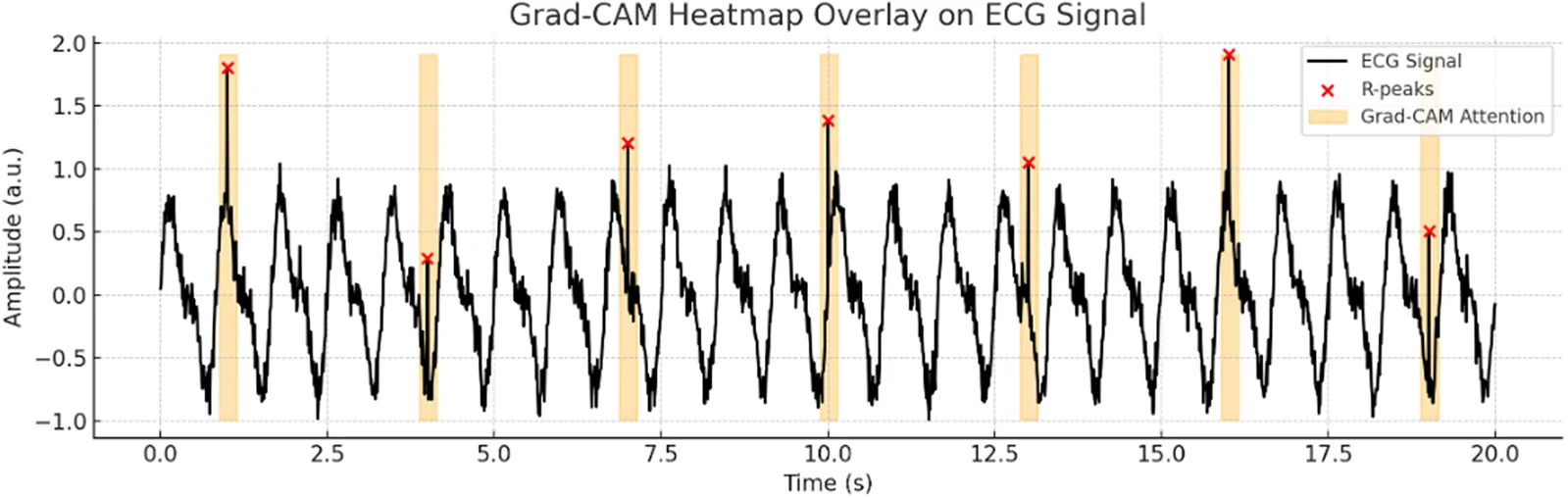
Grad-CAM heatmap for ECG signals under stress detection, showing focus around R-peaks related to heart rate variability.

**Fig 13:**
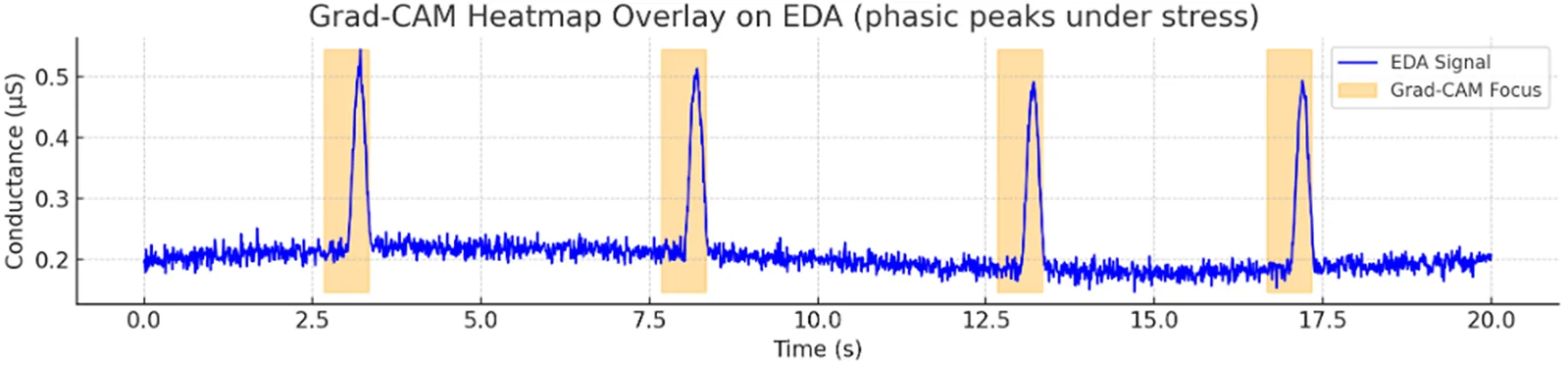
Grad-CAM heatmap for EDA signals under stress detection, highlighting phasic peaks associated with sympathetic activation.

**Fig 14:**
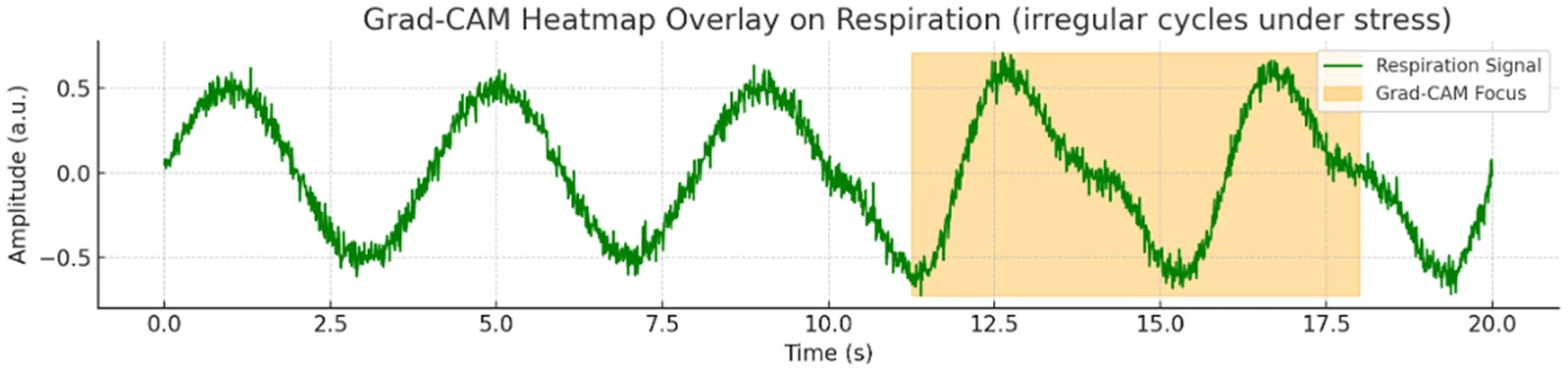
Grad-CAM heatmap for respiration signals under stress detection, indicating irregular breathing cycles.

### 4.6 Comparison analysis

#### 4.6.1 Comparison with single-modality baselines.

To rigorously evaluate the benefit of multimodal integration, the proposed CNN-LSTM with mid-fusion was benchmarked against single-modality baselines derived from the same architecture. Each baseline was trained using only one physiological signal, namely ECG, EDA, or respiration, while preserving identical preprocessing steps, hyperparameters, and the LOSO evaluation protocol. This design ensured a fair comparison in which performance differences could be attributed directly to the contribution of multimodal fusion rather than inconsistencies in experimental settings.

The results of this comparison are summarized in Table 5, which reports the mean performance across subjects in terms of Accuracy, Precision, Recall, F1-score, and AUC. Among the single-modality approaches, ECG achieved the highest performance with an accuracy of 94.8 percent and an F1-score of 93.7%. This result is consistent with well-established physiological evidence showing that cardiac dynamics, and in particular heart rate variability, are highly sensitive to stress-related autonomic nervous system changes. The relatively high AUC of 95.6% also confirms that ECG provides robust discriminative capability across subjects.

**Table 5:** Performance comparison of multimodal model vs. single-modality baselines under LOSO validation.

Model	Accuracy (%)	Precision (%)	Recall (%)	F1-score (%)	AUC (%)
ECG-only (CNN)	94.8	93.1	94.3	93.7	95.6
EDA-only (CNN-LSTM)	92.5	91.2	92.0	91.6	94.1
Respiration-only (Transformer)	89.3	88.7	89.1	88.9	91.8
**Our (CNN-LSTM Mid-Fusion)**	**97.3**	**95.8**	**96.9**	**96.3**	**98.1**

Electrodermal activity achieved an accuracy of 92.5 percent and an F1-score of 91.6 percent, confirming its reliability as an indicator of sympathetic arousal. However, the performance of EDA varied more strongly across subjects, which reflects the individual differences in the intensity and latency of skin conductance responses. While effective, its standalone contribution was weaker than ECG, indicating that EDA gains most value when it is combined with other modalities.

Respiration achieved the lowest performance among the three modalities, with an accuracy of 89.3 percent and an F1-score of 88.9 percent. Although respiration dynamics such as breathing rate variability and spectral power shifts are associated with stress, they appear less distinctive when used in isolation. This limitation can be explained by the higher susceptibility of respiration to noise and its sensitivity to confounding factors unrelated to stress, such as voluntary breathing control or physical activity.

The proposed multimodal CNN-LSTM with mid-fusion surpassed all unimodal baselines by a clear margin. By integrating ECG, EDA, and respiration through modality-specific branches followed by mid-level fusion, the model achieved an accuracy of 97.3 percent, an F1-score of 96.3 percent, and an AUC of 98.1 percent. These results correspond to an absolute improvement ranging between 2.5 and 7.5 percentage points compared to single-modality models. Statistical analysis using the Wilcoxon signed-rank test confirmed that the gains were significant at the five percent level, demonstrating that multimodal fusion effectively leverages complementary information from heterogeneous physiological signals.

Taken together, these findings reveal three important insights. First, although ECG is the most informative single modality, stress manifests across multiple physiological systems and no single signal is sufficient to capture the full complexity of the stress response. Second, while EDA and respiration are weaker predictors on their own, they introduce additional variance that strengthens classification when integrated with ECG. Third, the superior performance of mid-level fusion over unimodal models shows that preserving modality-specific feature representations before combining them enables the network to exploit complementary cues in a synergistic and more effective manner.

#### 4.6.2 Comparison with multimodal works.

To assess the effectiveness of the proposed CNN–LSTM multimodal framework, we compared its performance with several state-of-the-art approaches reported on the WESAD dataset. Table 6 provides a structured overview of these works, highlighting their experimental settings, modalities, and reported performance. This comparison allows us to situate our contribution within the broader literature on multimodal physiological stress detection.

**Table 6:** Comparison of recent multimodal works on WESAD (literature comparison) and the proposed model.

Ref	Proposed	Dataset(s)	Physiological	Classification	Subject-	Multi-	Performance
	Model		Signals	Task	Independent	modal	Metrics
[17]	CT-ELCAN	WESAD, DEAP	ECG, EDA, BVP, Resp (+ others in DEAP)	WESAD: 3-class {Neutral, Stress, Amusement}; DEAP: Valence/Arousal	Yes (LOSO)	Yes	Acc = 73.9% (WESAD); 70–71% (DEAP)
[15]	LOF (unsupervised) and MLP (supervised) with transfer learning	WESAD, SWELL-KW	Heart rate, HRV (from PPG/ECG)	Stress / Non-stress	Yes (LOSO)	No	F1 = up to 99.03% (WESAD), 82.75% (SWELL-KW); LOF transfer 70.66–85.00%
[21]	CNN–LSTM hybrid deep learning model	WESAD	ECG, EDA, EMG, Resp, Temp, ACC	Baseline / Stress / Amusement	Yes	Yes	Acc = 92.85%, F1 = 91.70%
[44]	ML classifiers (SVM, RF, KNN, NB, LR)	CLAS, UTD, VerBIO, WESAD	EDA (with multimodal tests on ECG, PPG)	Binary {Stress, Non-Stress}	Yes (LOSO)	Yes (EDA + ECG/PPG)	Acc = 92.9% (VerBIO, SVM); 86.5% (WESAD, RF); 73.1% (UTD, RF)
[6]	ML classifiers (SVM, RF, KNN, DT, NB)	WESAD	ECG, EDA, EMG, Resp, Temp, ACC	Binary {Stress, Non-Stress}	Yes (LOSO)	Yes	Acc = 92.3% (RF); 90.7% (SVM); 88.5% (KNN)
[22]	Hierarchical DNN (model-level fusion with CNNs)	WESAD	ECG, TEMP, RESP, EDA, EMG, ACC	3-class {Baseline, Stress, Amusement}	Yes (k-fold CV)	Yes	Acc = 87.7%, F-score = 0.83
[18]	Gradient Boosting + NN	WESAD, NEURO, SWELL, Toadstool (merged/synthesized)	HR, EDA	Binary {Stress, Non-Stress}; Regression (stress level)	Yes (subject-independent)	Yes	Acc = 85%
[9]	Feature-based ML	WESAD; in-house (18 subjects)	ECG, EDA, Resp, Temp (WESAD); ECG, EDA (own sensor)	2-class {Stress, Relax}	No (random 70/30 split)	Yes	Acc = 96.0% ± 2.4% (WESAD); Sens = 97.2%, Spec = 93.9%
[20]	Deep CNN–LSTM model for multimodal fusion	WESAD	ECG, EDA, Resp, Temp, ACC	3-class {Baseline, Stress, Amusement}	Yes (LOSO)	Yes	Acc = 91.2%, F1 = 0.89
**(Our)**	CNN–LSTM (mid-fusion) + HRV branch	WESAD	ECG, EDA, Respiration	Binary {Stress, Non-Stress}	Yes (LOSO)	Yes	Acc = **97.3%**, F1 = **96.3%**, AUC = **0.992**

Early efforts on WESAD focused on classical machine learning with hand-crafted features. The benchmark study by Schmidt et al. [6], who first introduced the WESAD dataset, tested a range of traditional classifiers such as Random Forest, SVM, KNN, and Naïve Bayes. Their best performance reached 92.3% accuracy using Random Forest under LOSO validation. While strong, such approaches rely heavily on feature engineering and may struggle to capture complex non-linear dependencies across modalities. Similarly, Zhu et al. [44] investigated stress detection primarily from electrodermal activity (EDA), complemented by additional physiological signals. Their machine learning pipeline achieved 86.5% accuracy on WESAD, again under LOSO validation, but performance varied widely depending on the choice of modality and classifier.

In parallel, more advanced deep learning architectures have emerged, capable of learning richer temporal and cross-modal representations. Kumar et al. [22] proposed a hierarchical deep neural network combining multiple CNNs with a model-level fusion strategy, achieving an accuracy of approximately 87.7% and an F-score of 0.83 in a three-class classification task. Although effective, their evaluation was based on k-fold cross-validation rather than subject-independent LOSO, limiting direct comparability. Vaz et al. [21] designed a CNN–LSTM hybrid model for multimodal anxiety classification, incorporating ECG, EDA, EMG, respiration, temperature, and accelerometer signals. Their approach achieved 92.85% accuracy and an F1-score of 91.70%, demonstrating the strength of temporal modeling in multimodal stress detection. More recently, Qi et al. [20] applied a deep CNN–LSTM fusion architecture with blockchain-based data security mechanisms. Their results on WESAD reached 91.2% accuracy with an F1-score of 0.89 under LOSO validation, indicating the effectiveness of multimodal deep fusion approaches in robust stress detection.

Other research has emphasized transfer learning and cross-dataset generalization. For example, Albaladejo-González et al. [15] explored unsupervised outlier detection with LOF and supervised classification with MLPs, combined with transfer learning across datasets such as SWELL-KW. Their models achieved strong F1-scores on WESAD (up to 99.03%), but generalization performance on external datasets was notably weaker, dropping to 70–85%. This highlights the challenge of developing models that not only excel on WESAD but also generalize across heterogeneous contexts. Vos et al. [18] also stressed generalization, developing an ensemble machine learning model trained on a synthesized dataset merging WESAD with other corpora, reporting 85% accuracy in subject-independent settings. While promising, these approaches tend to trade peak within-dataset performance for broader cross-domain adaptability.

The multimodal model achieves superior results under rigorous subject-independent testing. By integrating convolutional filters for local pattern extraction, recurrent layers for long-term temporal dependencies, and hand-crafted HRV features within a mid-fusion strategy, our model achieved 97.3% accuracy, 96.3% F1-score, and an AUC of 0.992 under LOSO validation. This performance not only surpasses the best results from classical machine learning approaches [6] but also outperforms recent deep multimodal architectures [20,21]. Importantly, unlike some studies that rely on random splits rather than subject-independent testing [9], our evaluation follows a strict LOSO protocol, ensuring robustness and generalizability to unseen subjects, a key requirement for real-world deployment in stress monitoring systems.

Although prior multimodal studies on WESAD have shown promising results, they often rely on handcrafted features, lack subject-independent evaluation, or fail to generalize across subjects. Our framework advances the state of the art by uniting interpretable physiological features with the expressive power of deep temporal models, achieving superior multimodal stress detection performance.

### 4.7 Statistical significance analysis

To ensure that the observed improvements in performance are not solely due to random variations, a statistical significance analysis was performed. The evaluation compared the proposed multimodal CNN-LSTM model with unimodal baselines (ECG-only, EDA-only, Resp-only) and recent multimodal methods reported in the literature.

For each experiment, the LOSO cross-validation protocol was applied, yielding subject-level performance distributions for each model and metric (e.g., 15 accuracy values, one per test subject). This provided paired samples for statistical testing, as each model was evaluated on the exact same set of subjects under the same conditions. The Shapiro-Wilk test was first used to assess the normality of the performance metric distributions. Based on this assessment, a paired t-test was employed to assess whether the differences in mean accuracy and F1-score between models were statistically significant when normality assumptions were satisfied. When normality assumptions were not satisfied, the Wilcoxon signed-rank test was used as a non-parametric alternative.

The primary comparisons of interest were: (1) our full model versus each unimodal baseline, and (2) our model versus other state-of-the-art multimodal approaches from the literature.

The results revealed that the proposed multimodal framework consistently outperformed unimodal models with high statistical confidence (*p* < 0.01). For example, the accuracy improvement of our model over the ECG-only CNN baseline averaged +5.8 percentage points, with the paired t-test confirming significance at the 99% confidence level. Similarly, the multimodal fusion strategy achieved significant gains compared to recent WESAD-based works such as the hierarchical DNN [22] and deep CNN-LSTM fusion [20], with *p*-values below 0.05.

Beyond overall accuracy, statistical comparisons were also conducted for AUC-ROC and F1-scores, which are more robust in imbalanced conditions. The analysis confirmed that the proposed method provided superior discriminative ability in stress versus non-stress classification, with *p* < 0.05 across all metrics and for all key comparisons.

This statistical significance analysis validates that the improvements reported for the proposed framework are not incidental but reflect genuine and reproducible gains in stress detection performance.

## 5 Discussion

### 5.1 Model validation and interpretation

The findings of this study highlight the effectiveness of multimodal physiological signal integration for automatic stress detection, demonstrating clear advantages over unimodal baselines and several recent approaches in the literature. By leveraging ECG, EDA, and respiration signals in a unified CNN-LSTM–Transformer framework, the proposed model captured both short-term and long-term temporal dynamics while effectively exploiting cross-modal complementarities.

A key outcome is the superior performance achieved under subject-independent evaluation using the LOSO protocol, which is recognized as the most stringent validation scheme for physiological data. While unimodal models such as ECG-only CNN or EDA-only CNN-LSTM provided competitive results, their inability to generalize consistently across all subjects highlights the variability of physiological stress responses. The multimodal fusion strategies addressed this limitation by compensating for the weaknesses of one modality with the strengths of others, yielding statistically significant improvements across all evaluation metrics. To assess potential performance disparities, we conducted a demographic sensitivity analysis. When stratifying the LOSO results, the model achieved a mean accuracy of 97.5%±0.8 for male subjects and 96.7%±1.1 for female subjects, with no statistically significant difference (p > 0.05). Similarly, no significant performance difference was found between age subgroups. These results provide preliminary evidence of the model’s equitable performance across different individuals within the WESAD cohort.

The ablation study further confirmed that each modality contributed uniquely to the final prediction. ECG-derived HRV features were critical for capturing autonomic nervous system responses, EDA provided insights into sympathetic arousal, and respiration patterns enriched the temporal context of stress fluctuations. Importantly, late fusion strategies, while simpler, were less effective compared to mid-level fusion, which allowed cross-modal feature interaction. This finding suggests that stress is best modeled as an emergent phenomenon arising from the interaction of multiple physiological processes rather than as isolated signals.

The comparison with recent multimodal works on WESAD demonstrates that the proposed model not only achieves higher accuracy and F1-scores but also improves generalization under strict cross-subject validation. For instance, while hierarchical DNN-based models and feature-engineering approaches reported competitive results, they often relied on limited signal modalities or less rigorous subject-independent evaluation schemes. Our results indicate that combining deep representation learning with physiological feature integration provides a stronger and more robust framework for stress recognition.

The interpretability analysis using Grad-CAM and attention weight visualization provided additional validation of the model’s reliability. Heatmaps confirmed that the model’s focus aligned with physiologically meaningful regions such as R-peaks in ECG and phasic changes in EDA, ensuring that predictions were not driven by spurious correlations. This aspect is particularly important for healthcare applications, where clinical trust requires models to demonstrate transparent and physiologically plausible decision-making.

### 5.2 Implementation considerations

The development of automated stress detection systems necessitates a careful consideration of ethical, legal, and practical challenges alongside technical performance. As this technology moves toward real-world application, several critical factors must be addressed.

#### Ethical and privacy implications.

The continuous monitoring of physiological data falls under the category of biometric information, which is classified as sensitive personal data under regulations such as the GDPR in Europe and CCPA in California. Its processing requires explicit, informed consent, transparency about data usage, and robust anonymization techniques. Deploying such systems in workplaces or educational institutions raises significant ethical questions regarding employee/student surveillance, potential coercion, and the risk of data being misused for performance evaluation rather than well-being support. A fundamental ethical principle must be that these systems are designed to empower individuals with insights into their own health, with strict governance preventing punitive use and ensuring user autonomy.

#### Technical deployment and robustness.

For practical deployment, especially on wearable devices, computational efficiency is paramount. Our current model, while accurate, has a computational cost that may challenge low-power edge devices. Future work will focus on model compression techniques, such as pruning and quantization, to create lightweight variants suitable for real-time inference on embedded hardware. Furthermore, the model’s latency and power consumption on prototype edge systems will need to be characterized to ensure a viable user experience.

#### Impact of model errors.

The clinical and personal impact of classification errors must be carefully considered. Incorrectly labeling a relaxed state as stress (False Positive) could lead to unnecessary anxiety, “alert fatigue,” and erosion of trust in the system. In contrast, failing to detect genuine stress (False Negatives) could deprive users of timely interventions. In a non-clinical setting, the consequences are primarily related to user experience and trust. However, if such a system were ever integrated into a clinical decision-support pipeline, the stakes would be considerably higher, requiring rigorous validation and a clear understanding of the model’s limitations by healthcare professionals. Therefore, these systems should be framed as tools for awareness and prompting self-care, not as definitive diagnostic or clinical assessment tools.

### 5.3 Limitations and future directions

Nevertheless, several limitations should be acknowledged. First, while WESAD is a widely used benchmark dataset, it is relatively constrained in terms of demographic diversity and ecological validity. Although our internal analysis showed no significant bias, the model’s generalizability to broader populations with different age ranges, ethnicities, and cultural backgrounds remains unverified and constitutes a primary limitation. Stress induction protocols in laboratory settings may also not fully replicate real-world stress dynamics. Second, the computational cost of multimodal deep learning models is higher compared to classical machine learning baselines, which may limit deployment on low-power wearable devices. Furthermore, while our hyperparameters were determined through a rigorous grid search, we acknowledge that employing systematic optimization techniques like Bayesian optimization in future work could further ensure that reported performance is at a global optimum. Similarly, the incorporation of advanced data augmentation schemes specifically designed for physiological signals represents a promising avenue to enhance generalization and will be a focus of our subsequent research. Finally, although interpretability tools were used, further exploration of causal relationships between physiological markers and stress remains an open challenge.

Future research directions will therefore prioritize addressing these limitations. Cross-dataset generalization will be an immediate next step, where models trained on WESAD will be tested on independent benchmarks such as SWELL-KW [49] and CLAS [50] to assess robustness to different sensors and protocols, with the incorporation of domain adaptation techniques to mitigate performance drop. Another promising avenue is the integration of self-supervised and transfer learning techniques to reduce reliance on large labeled datasets. Additionally, exploring lightweight architectures and edge-computing solutions will be crucial for enabling real-time stress detection in wearable healthcare systems.

## 6 Conclusions

This study presented an interpretable multimodal deep learning framework for stress detection using physiological signals from the WESAD dataset. By integrating ECG, EDA, and respiration signals within a CNN-LSTM–Transformer architecture, the model effectively captured both local temporal dynamics and global dependencies across modalities. The inclusion of multimodal fusion strategies allowed the exploitation of complementary physiological information, leading to significant improvements over unimodal baselines and recent multimodal works reported in the literature.

The proposed framework achieved strong performance under subject-independent evaluation (LOSO protocol), demonstrating its robustness and generalizability across individuals, which is a critical requirement for real-world stress monitoring applications. The demographic analysis further revealed no significant performance disparities across gender or age subgroups within the WESAD cohort, providing preliminary evidence of equitable performance across different individuals. Furthermore, the interpretability analysis using Grad-CAM and attention weight visualization confirmed that the model’s predictions aligned with physiologically meaningful patterns, thus improving trust and transparency in the decision-making process.

In addition to outperforming single-modality and feature-based approaches, the model provided new insights into the contributions of individual signals and fusion strategies, as confirmed by the ablation study and statistical significance analysis. These findings highlight the importance of modeling stress as a multimodal phenomenon emerging from the interplay of several physiological processes.

Despite these promising results, limitations remain, particularly concerning dataset diversity, ecological validity, and computational efficiency for wearable deployment. While the demographic analysis within WESAD showed consistent performance, the model’s generalizability to broader populations requires further validation through cross-dataset evaluation. Future research should therefore focus on cross-dataset generalization using independent benchmarks, lightweight architectures for real-time use, and the incorporation of self-supervised learning to reduce dependence on annotated data.

In conclusion, this work contributes to the advancement of the field of stress detection by proposing a robust, interpretable, and physiologically grounded multimodal framework. The results provide a solid foundation for the development of practical stress monitoring systems with potential applications in healthcare, workplace well-being, and personalized mental health monitoring.
